# Highly selective inhibition of histone demethylases by *de novo* macrocyclic peptides

**DOI:** 10.1038/ncomms14773

**Published:** 2017-04-06

**Authors:** Akane Kawamura, Martin Münzel, Tatsuya Kojima, Clarence Yapp, Bhaskar Bhushan, Yuki Goto, Anthony Tumber, Takayuki Katoh, Oliver N. F. King, Toby Passioura, Louise J. Walport, Stephanie B. Hatch, Sarah Madden, Susanne Müller, Paul E. Brennan, Rasheduzzaman Chowdhury, Richard J. Hopkinson, Hiroaki Suga, Christopher J. Schofield

**Affiliations:** 1 Chemistry Research Laboratory, Department of Chemistry, University of Oxford, 12 Mansfield Road, Oxford OX1 3TA, UK; 2 Division of Cardiovascular Medicine, Radcliffe Department of Medicine, Wellcome Trust Centre for Human Genetics, Roosevelt Drive, Oxford OX3 7BN, UK; 3 Department of Chemistry, Graduate School of Science, The University of Tokyo, 7-3-1 Hongo, Bunkyo-Ku, Tokyo 113-0033, Japan; 4 Structural Genomics Consortium, University of Oxford, Old Road Campus Roosevelt Drive, Headington OX3 7DQ, UK; 5 Nuffield Department of Medicine, Target Discovery Institute, University of Oxford, Roosevelt Drive, Oxford OX3 7FZ, UK; 6 JST, CREST, 7-3-1 Hongo, Bunkyo-Ku, Tokyo, 113-0033, Japan

## Abstract

The JmjC histone demethylases (KDMs) are linked to tumour cell proliferation and are current cancer targets; however, very few highly selective inhibitors for these are available. Here we report cyclic peptide inhibitors of the KDM4A-C with selectivity over other KDMs/2OG oxygenases, including closely related KDM4D/E isoforms. Crystal structures and biochemical analyses of one of the inhibitors (CP2) with KDM4A reveals that CP2 binds differently to, but competes with, histone substrates in the active site. Substitution of the active site binding arginine of CP2 to *N*-ɛ-trimethyl-lysine or methylated arginine results in cyclic peptide substrates, indicating that KDM4s may act on non-histone substrates. Targeted modifications to CP2 based on crystallographic and mass spectrometry analyses results in variants with greater proteolytic robustness. Peptide dosing in cells manifests KDM4A target stabilization. Although further development is required to optimize cellular activity, the results reveal the feasibility of highly selective non-metal chelating, substrate-competitive inhibitors of the JmjC KDMs.

## Abstract (web summary)

JmjC histone demethylases (KDMs) are cancer targets due to their links to cell proliferation, but selective inhibition remains a challenge. Here the authors identify potent inhibitors of KDM4A-C—via *in vitro* selection from a vast library of cyclic peptides—that show selectivity over other KDMs.

Eukaryotic gene expression is substantially regulated by a complex set of covalent modifications to DNA and histones^1^,^2^. Aberrant histone modifications are associated with diseases, including cancer, inflammatory disorders, neurological and cardiovascular diseases^3^,^4^. The multi-domain proteins that read and write posttranslational histone modifications are often mutated/dysregulated in such diseases. Structural similarities between individual domains of these ‘epigenetic' proteins make the identification of potent and selective inhibitors of these proteins challenging.

Histone lysine methylation is dynamically regulated by methyltransferases and demethylases (KDMs). Two families of human KDMs catalyse the removal of methyl groups from methylated lysines on histone tails in a sequence- and methylation-state-dependent manner^5^; the flavin-dependent lysine-specific demethylases (KDM1/LSD)^6^ and the JmjC-domain containing KDMs (JmjC-KDMs), which are Fe(II) and 2-oxoglutarate (2OG)-dependent oxygenases (KDM2–7)^7^,^8^. Only a few JmjC-KDM inhibitors are reported, the majority of which operate via active-site metal chelation^9^. Achieving both selectivity and potency for specific JmjC-KDMs remains a major challenge^9^.

Natural product screening is an attractive method for identifying inhibitor hits and leads. However, natural product-based structure activity studies are often limited by difficulties in synthesis and/or systematically manipulating the output of biosynthesis. An alternative approach, Random nonstandard Peptides Integrated Discovery (RaPID) system has been developed, which involves efficient selection using ribosomally expressed ‘natural product'-inspired macrocycles^10^,^11^. *De novo* macrocycles that tightly bind to target proteins can be efficiently selected from the >10^12^ members of the library; derivatives of the initial hits are then chemically synthesized for structural optimizaion^11^,^12^,^13^.

We describe the use of the RaPID methodology for discovery of highly selective and potent cyclic peptide inhibitors of KDMs, which, after structure- and activity-guided modifications, show evidence of on-target engagement in cells. We targeted the KDM4 subfamily, which represent biomedically attractive but challenging targets. Although the catalytic domains (JmjC-domain) and active sites are highly conserved, all KDM4s remove the repressive H3K9me3 mark, but only KDM4A-C are additionally capable of demethylating the activating H3K36me3 mark^14^,^15^. Intra-subfamily selective inhibitors will be useful tools to dissect the roles of the opposing histone modifications and of the KDM4 isoforms in disease.

## Results

### Identification of potent KDM4A-C-selective cyclic peptides

**Figure 1:**
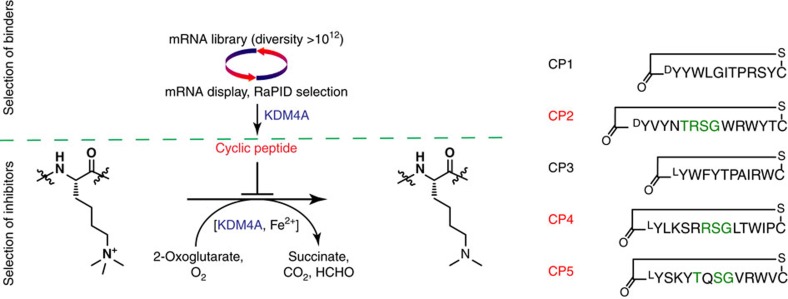
Development of KDM4A cyclic peptide inhibitors. Cyclic peptide binders of KDM4A were selected using the RaPID system (Supplementary Fig. 1). The hit peptide sequences (CP1–CP5) were synthesized and further tested in enzymatic assays. Peptides were cyclized by a thioether formation.

A messenger RNA template library was designed with the general form AUG-(NNK)_4–12_-UGC, where the AUG start codon was reassigned from Met to either *N*-chloroacetyl-L-Tyr or *N*-chloroacetyl-D-Tyr, which induce cyclization of the translated peptides through reaction with an appropriately positioned cysteine (UGC) downstream of the random sequence to yield lantibiotic-like thioether rings^16^,^17^. The selection used two thioether-macrocycle libraries (^L^Tyr and ^D^Tyr libraries)^12^ with screening against the catalytic domain of His-tagged KDM4A (residues 1–359) (Fig. 1 and Supplementary Fig. 1a). Enriched complementary DNA pools from both libraries were cloned and analysed. Out of 21 (^D^Tyr-library) and 23 (^L^Tyr-library) clones sequenced (Supplementary Fig. 1b,c), five cyclic sequences (CP1–5, 11–14 residues in length) were selected for further analysis based on hit frequency and similarity; these were synthesized by solid-phase peptide synthesis. Catalytic inhibition of KDM4A/C by the selected peptides was tested using a luminescence-based AlphaScreen activity assay^18^. From the KDM4A screen, two cyclic peptides (CP2 and CP4) demonstrated potencies of half-maximal inhibitory concentration (IC_50_)<50 nM against KDM4A (Table 1); CP5 was less potent (IC_50_<500 nM). The other two hits (CP1 and CP3) were much less effective (IC_50_>100 μM).

Clear selectivity (that is, >160-fold by IC_50_) of all inhibitory CPs (CP2, CP4 and CP5) was observed for KDM4 over representatives from four other human JmjC-demethylase families and two related 2OG oxygenases, prolyl hydroxylase domain 2 and factor inhibiting HIF, as well as a flavin-dependent KDM (KDM1A), indicating that these CPs are exceptionally selective (Table 1). Intra-subfamily selectivity was then investigated by determining IC_50_ values across all KDM4 subfamily members. CP2 and CP4 displayed high potency (IC_50_<50 nM) against KDM4A–C; importantly, they were much less active against KDM4D–E, revealing intra-subfamily selectivity of >100-fold and that the CPs are therefore even capable of distinguishing the subtle differences between KDM4A–C and KDM4D/E. Binding studies demonstrated that CP2, CP4 and CP5 are tight binders of KDM4A with slow on and off rates; in contrast, the histone H3K9 peptide (KDM4A substrate) exhibits substantially faster on/off rates and weaker binding affinity (*K*_d_>1,000-fold; Table 1 and Supplementary Fig. 2a,b). Kinetic analyses with CP2 revealed it is a competitive inhibitor with respect to H3K9me3 (Supplementary Fig. 2c). CP2 also inhibits the demethylation activity of full-length KDM4A (residues 1–1,064) purified from HEK293T cells (Supplementary Fig. 3), indicating CP2 can inhibit KDM4A when other domains (for example, PHD/tudor domains) are present.

### CP2 uniquely binds in the KDM4A substrate-binding pocket

**Figure 2:**
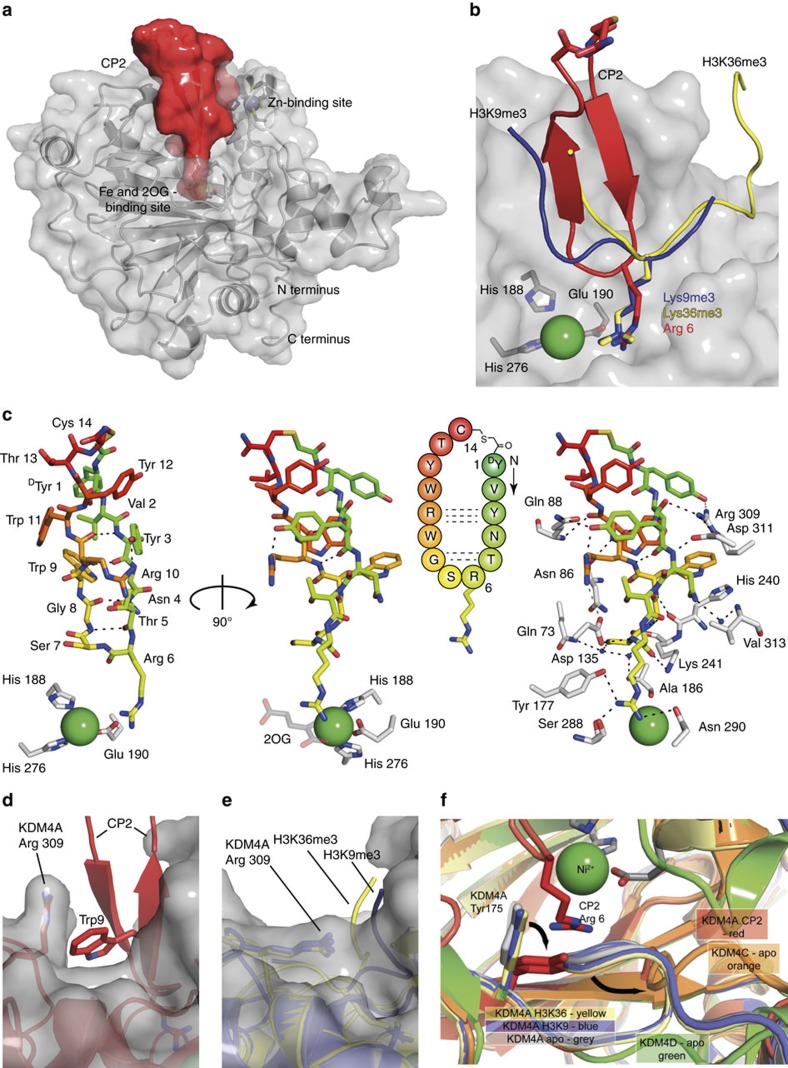
Cyclic peptide (CP2) occupies the substrate binding site of KDM4A. (**a**) Space-filling view from a crystal structure of KDM4A complexed with CP2. (**b**) Overlay of CP2 with the backbones of the histone substrates (H3K9me3 (PDB 2OQ6) and H3K36me3 (PDB 2P5B)) of KDM4A. The sidechain of Arg6 projects towards the metal as observed with the Kme3 substrate sidechain. (**c**) CP2 adopts a twisted β-sheet fold with a type-1 β-turn at the active site and engages in an extensive hydrogen bond network (Supplementary Fig. 4) and binds in the histone substrate binding site. (**d**–**f**) CP2 binding shows distinct differences in some of the side-chain orientations of KDM4A to that induced by histone peptide binding. (**d**) Trp9 in CP2 induces movement of Arg309 in KDM4A, relative to the (**e**) histone peptide-bound KDM4A structures. (**f**) Movement of Tyr 175 in KDM4A is observed when bound to CP2, relative to the histone peptide bound structures of KDM4A, and induces a shift in the adjacent loop. This loop orientation is similar to that observed in the KDM4C (apo, orange (PDB 2XML)) structure. Ni(II) is substituted for Fe(II) in the KDM4A crystals.

To investigate the structural basis of the unprecedented selectivity of the cyclic peptides, we crystallized CP2 with KDM4A (2.7 Å resolution, Fig. 2a). Interestingly, the structure reveals that CP2 occupies the histone-binding groove of KDM4A, but not the 2OG-binding pocket, the latter of which has been exploited for most reported 2OG oxygenase, including JmjC KDM, inhibitors. Moreover, CP2 exhibits a binding mode, which partially overlaps, but which is clearly distinct from those observed for H3K9me(*n*) and H3K36me(*n*) histone substrates (Fig. 2b). Analysis of the inhibitor conformation revealed that CP2 adopts a distorted β-sheet fold with two turns; a type 1 β-turn (Thr5-Gly8) is closest of the two β-turns to the active site metal (Fig. 2c). CP2 engages in multiple hydrogen bonds with KDM4A, many of which are not observed in the reported structures of KDM4A-H3K9me3 (PDB 2OQ6) and KDM4A-H3K36me3 enzyme substrate complexes (PDB 2P5B)^19^. Strikingly, the structure reveals Arg6-CP2 bound in the sub-pocket usually occupied by the extended trimethyl-lysine side chain of the H3K9 and K36 substrate residues. In addition to mimicking the positive charge of the lysine, the guanidino group of Arg6-CP2 is positioned to make hydrogen bonds with KDM4A residues (Tyr177, Ser288 and Asn290; Supplementary Fig. 4a,b). Two KDM4A residues in the CP2-complexed structure (Tyr175 and Arg309) move, relative to uncomplexed KDM4A, to open pockets that accommodate CP2, suggesting flexibility in these regions. The Arg309 side chain rotates by approximately 90° relative to the H3K9me3/H3K36me3 bound KDM4A structures to accommodate Trp9-CP2 (Fig. 2d,e). Tyr175 also shifts by approximately 90° to accommodate Arg6-CP2 (Fig. 2f). This movement relocates an adjacent loop (between α6 and DSBH βI (β7), residues 161–173), which resembles a conformation found in KDM4C(apo) structure (Fig. 2f). Other notable conformational changes in the KDM4A residues induced by CP2 binding include at Lys241 and Asp311 (Supplementary Fig. 5).

Although the linearized sequence of CP2 maintains modest activity against KDM4A, its IC_50_ is four fold higher than that of CP2, probably due to the increased conformational freedom of the linear sequence (Table 2, peptide 2). However, changing the stereochemistry of the ‘cyclizing' amino-terminal tyrosine (D/L-Tyr) does not affect the potency of CP2 (Table 2, peptide 3); this may reflect the lack of interactions that CP2 makes with KDM4A in the regions surrounding the thioether bond (Tyr1, Val2 and Tyr12-Cys14) as observed crystallographically (Fig. 2c and Supplementary Fig. 4a,b).

Analysis of the polar contacts in the KDM4A-CP2 structure provides a structural basis for the selectivity of the cyclic peptides for KDM4A-C over D/E (Supplementary Fig. 4c). Despite the sequence and structural similarity in the catalytic domains between KDM4A and KDM4D (77%)^20^, 5 of the 14 residues directly interacting with CP2 in KDM4A–C (Asn86, Gln88, Ser288, Arg309 and Asp311) are different in KDM4D/E. Interestingly, three of the five residues (Asn86, Gln88 and Arg309) coincide with those identified as important for the lack of activity of KDM4D/E on H3K36me3 (ref. 20).

### Arg6-CP2 is a key residue for KDM4A binding

**Figure 3:**
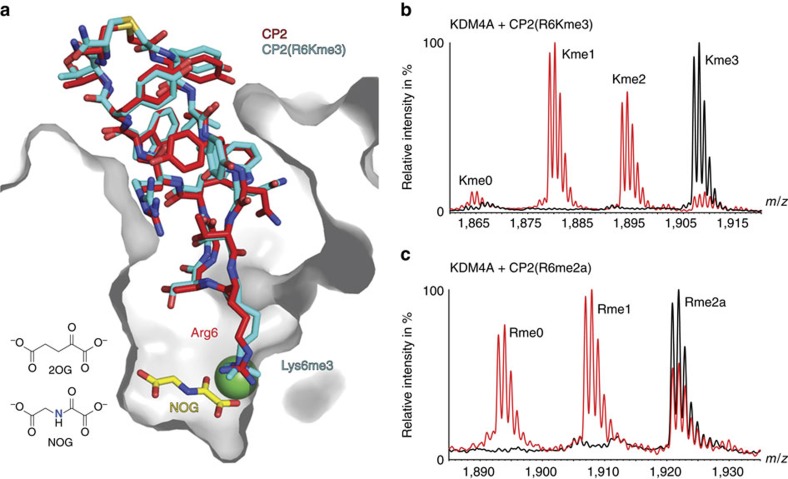
KDM4A can demethylate methylated lysine and arginine containing non-histone sequences. (**a**) Overlay of views from crystal structures of KDM4A with CP2 (red) and CP2(R6Kme3).NOG (cyan). It is noteworthy that the binding site of NOG, an inactive 2OG analogue, is distinct from the binding site of CPs. (**b**) CP2(R6Kme3) (peptide 9) and (**c**) CP2(R6me2a) (peptide 10) are substrates of KDM4A. KDM4A_1-359_ (2 μM) was incubated with CP2 variant (10 μM) in the presence of 2OG (100 μM), Fe(II) (10 μM) and ascorbate (100 μM) for 2 h at 37 °C. The reaction product was analysed using matrix-assisted laser desorption/ionization–time of flight MS. Reactions containing enzymes are in red, no enzyme peptide controls are in black.

To investigate the role of Arg6 of CP2, which binds in a similar manner to the H3Kme(*n*) residue, we substituted it with other residues (Table 2 and Supplementary Fig. 6); substitution with alanine (peptide 4) or with hydrophobic/uncharged side chains including *N*-ɛ-acetyl lysine and citrulline (peptides 5, 6 and 7) leads to loss of potency (IC_50_>2,400 nM for KDM4A). Inhibition of activity is regained upon substitution with positively charged residues (lysine (peptide 8)/*N-ɛ*-trimethyllysine (peptide 9), IC_50_=24 and 12 nM, respectively, for KDM4A). The relevance of the crystallographically observed orientation of Arg6 in solution was explored using the trimethyl-lysine CP2 variant (peptide 9). Despite the lack of similarity between CP2 and the histone substrates, *N*-ɛ-trimethyl-lysine-CP2 (R6Kme3) was efficiently demethylated by KDM4A to Kme2/Kme1/Kme0 (Fig. 3b). A co-crystal structure of CP2(R6Kme3) in complex with *N*-oxalylglycine (NOG, an inactive form of 2OG) and KDM4A supports the productive binding conformation of the CP2(R6Kme3) (Fig. 3a); the acceptance of CP2(R6Kme3) as a KDM4A substrate unequivocally demonstrates that the CP2 series does not compete with 2OG. Notably, KDM4A was also able to demethylate methylated Arg6 (CP2(R6me2a), peptide 10) to the non-methylated, inhibitory CP2 sequence (Fig. 3c). The different sequences and binding modes between the well-established histone substrates (H3K9me3/K36me3) and CP2 suggests that KDM4s might be more promiscuous in their substrate selectivity and binding mode than presently perceived.

### Structure-guided modifications improve stability in cells

We then investigated the utility of CP2 as a cellular probe. Strain-promoted azide-alkyne cycloaddition was used to generate CP2 derivatives with a fluorescein dye (CP2(Fl)) (Supplementary Fig. 6a)^21^,^22^. Time-dependent increases in intracellular fluorescence were observed in HeLa cells dosed with CP2(Fl), indicating cellular uptake (Supplementary Fig. 7). The fluorescence signal was observed to accumulate outside the nucleus (>10 h), possibly indicating peptide degradation. Cellular thermal shift assays (CETSA)^23^ were then used to investigate intracellular target engagement of CP2 in cells. U2OS cells ectopically expressing FLAG-tagged KDM4A_1–1,064_ were dosed with CPs for 20 h, harvested, washed and the intact cells were subjected to a temperature gradient. CETSA melt curves were determined for KDM4A and actin by western blot analysis. The melting temperature (*T*_m_) for FLAG-KDM4A was shifted from 47 °C (dimethyl sulfoxide, DMSO) to 59 °C with CP2(T13Z) (the precursor to the labeled CP2(Fl), peptide 25) (Fig. 5b and Supplementary Fig. 6b). Isothermal dose–response function (ITDRF) at 55 °C revealed FLAG-KDM4A stabilization by CP2 in a dose-dependent manner (EC_50_∼1 μM), supporting intracellular target engagement of the inhibitor (Fig. 5c). Correlating with the selective inhibition of CP2 for isolated KDM4A-C, FLAG-KDM4E was not stabilized under the same CETSA conditions (Supplementary Fig. 8). However, despite apparent intracellular KDM4A engagement, CP2-treated cells did not have any apparent effect on the global histone methylation levels at H3K9me3 and H3K36me3 mark (Supplementary Fig. 12).

Addition of a poly-arginine sequence to CP2 aimed at enhancing the cellular uptake (CP2(polyR), peptide 11) unexpectedly increased the potency against KDM4s *in vitro* (KDM4A IC_50_=1.8 nM, KDM4C IC_50_=0.8 nM; Table 2). Interestingly, polyR alone is a potent KDM4A inhibitor (IC_50_=40 nM); thus, the increased potency of CP2(polyR) is likely to be a combined effect of the two inhibitory elements. However, although cytotoxicity was observed at high concentrations (>3 μM) with significant reduction in cell numbers, no inhibition of cellular KDM4A demethylase activity by CP2(polyR) was detected (Supplementary Figs 11 and 13). An analogous phenomenon has been previously reported with disulphide linked cyclic peptide generated against KDM4C using phage display^24^; the potency of a proposed allosteric binding cyclic peptide inhibitor (IC_50_=52 μM) was improved to IC_50_=0.6 μM on addition of a poly arginine/lysine (TAT) tag, but no cell activity was observed.^25^

**Figure 4:**
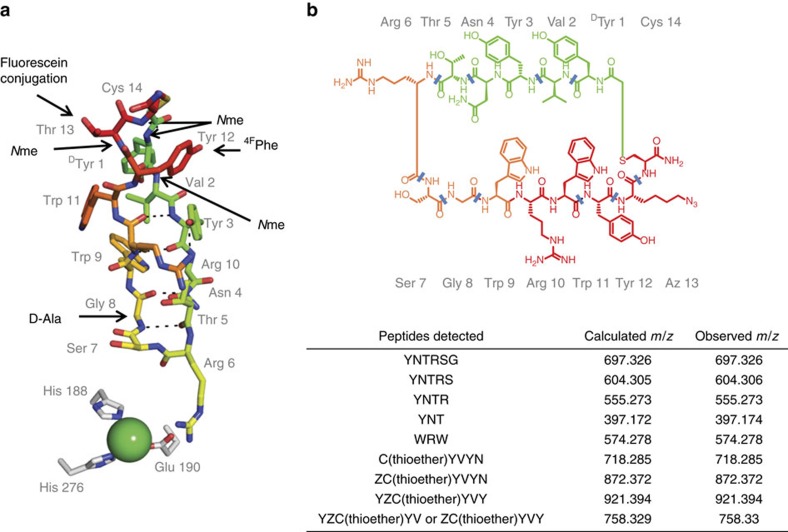
Crystallography and mass spectrometry guided modifications of CP2. (**a**) Sites of modifications were made based on the crystal structure and MS degradation analysis. (**b**) MS analysis of degradation fragments of CP2(T13Z) observed on incubation with cell lysate. Cleavage sites are indicated as blue lines.

**Figure 5:**
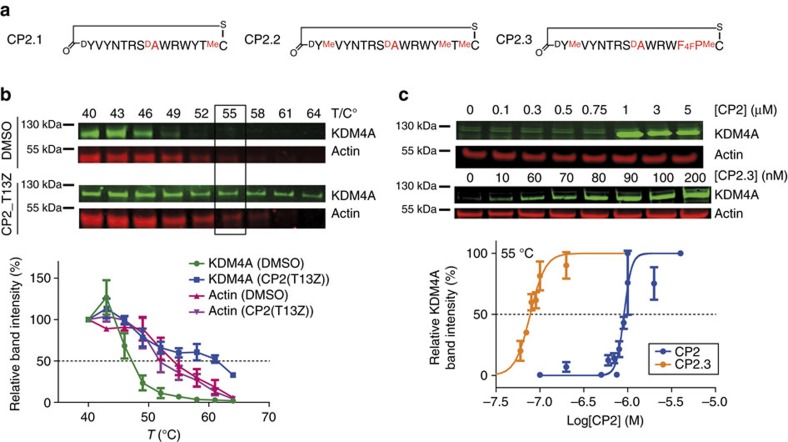
CP2 derivatives stabilize KDM4A and alter histone methylation status in cells. (**a**) Sequences of peptides with modifications on CP2 (CP2.1, CP2.2 and CP2.3) used for cellular assays. (**b**,**c**) KDM4A stability is enhanced for CP2-treated cells in CETSA. (**b**) CETSA melting curves for Flag-KDM4A in U2OS cells with and without CP2(T13Z) treatment (0.5 μM). The actin *T*_m_ was 55 °C for treated and untreated cells. (**c**) Isothermal dose–response titration CETSAs of dosed U2OS cells at 55 °C demonstrate that both CP2 and CP2.3 stabilize KDM4A in a dose-dependent manner. Average±s.e.m. are shown (*n*>3, biological). Representative western blotting figures are shown.

We then modified CP2 by backbone amide *N*-methylation, which is reported to improve peptide stability and cellular uptake.^25^ Guided by the CP2-KDM4A crystal structure (Fig. 2), we synthesized mono *N*-methylated variants at backbone amide positions not engaging in critical hydrogen bonding interactions with KDM4A and within the CP2 β-sheet. The selected residues, Cys14, Thr13, Val2 and Tyr1, were mainly in the non-interacting, thioether-linkage region of CP2 (Table 2 and Fig. 4a; peptides 13–16). Mass spectrometric (MS) analysis of CP2 degradation patterns after incubation in cell lysates confirmed that these sites are prone to proteolysis (Fig. 4b and Supplementary Fig. 9). D-Ala was also substituted for Gly8 of CP2 with the aim of improving proteolytic stability, as the crystal structure predicted this substitution would be tolerated and hydrolysis at this site was observed by MS (Fig. 4b). 4-Fluorophenylalanine was further incorporated with the aim of improving cellular uptake by increasing hydrophobicity (peptides 17 and 18). Although some of the CP2 modifications reduced activity, in general the structure-guided modifications were well tolerated. Combinations of tolerated modifications were prepared (named as CP2.1, CP2.2 and CP2.3, peptides 19–21, respectively) (Table 2, Fig. 5a and Supplementary Fig. 6); Potency and selectivity for KDM4A over other KDM families were maintained for these modified CPs with isolated enzymes. (Supplementary Table 1). Interestingly, the inhibitory effect of CP2.3 against KDM4B was much weaker than for CP2, but was retained for KDM4A/C. CP2.3 demonstrated greater stability in HeLa cell extracts (*t*_1/2_∼5 h) compared with CP2 (*t*_1/2_∼1 h), as determined by MS (Supplementary Fig. 10). Furthermore, the modified CP2.3 was able to stabilize FLAG-KDM4A as observed in CETSA, at concentrations lower than the unmodified CP2 (EC_50_<100 nM) (Fig. 5c), suggesting that the target engagement was maintained on modification and that peptide cellular stability was increased.

We tested CP2.1, CP2.2 and CP2.3 for KDM inhibition in HeLa cells by immunofluorescence analysis of histone methylation marks (72 h treatment). Although application of the CP2 parent gave no discernible changes, treatment with CP2.1–3 manifested increases in nuclear H3K9me3/H3K36me3 staining over the 10–20 μM inhibitor range (Supplementary Fig. 12a–g). No changes were observed in the apparent H3K9me3 levels when cells were treated with the inactive CP2.3(R6A) peptide (peptide 22; Table 2 and Supplementary Fig. 12f). A significant reduction in cell numbers was observed in HeLa cells treated with CP2.1–3 (>10 μM) (Supplementary Fig. 12e). As hypermethylation can correlate with cellular stress/toxicity, it is unclear whether the observed hypermethylation is due to on-target KDM4 inhibition or cytotoxic effects, or a combination of these. However, dosing HeLa cells ectopically overexpressing FLAG-KDM4A (wild-type or catalytic inactive mutant H188A) with CP2.3 for 24 h showed no significant changes in H3K9me3 levels (Supplementary Fig. 13) at non-cytotoxic concentration range (<10 μM). Thus, the biological relevance of the cellular observations of changes in global histone methylation status following treatment with the KDM inhibitors should be treated with caution.

## Discussion

In conclusion, we have used *in vitro* selection from a ribosomally synthesized library of cyclic peptides to identify natural product-like inhibitors of KDM4A-C, which act via a previously unidentified binding mode and which have unprecedented selectivity and potency. The RaPID display approach is substantially more efficient than traditional medicinal chemistry and is likely to be of widespread utility in target-based probe discovery. The method is well-suited to identify new inhibitor binding modes, as revealed by the structures of KDM4A complexed with CP2 and CP(R6Kme3), and associated biochemical results. The binding mode of CP2 is distinct from reported KDM4C peptide inhibitors (with IC_50_ values in the μM range) based on the outputs of a phage display library screen, which probably do not bind at the active site (structural studies are not available)^24^. The sequence of CP2 is clearly distinct from that of well-characterized histone substrates for KDM4A–C. The importance of the anchoring residue Arg6 within the CP2 sequence for potent KDM4A inhibition, suggests that arginine residues can compete with methylated lysines binding to KDM4A. This is significant, given the recent findings that some, but not all, JmjC-KDMs, including some KDM4 subfamily members, can also act as *N*^*ɛ*^*-*methyl arginine demethylases^26^. KDM4A can both catalyse the demethylation of CP2(R6Kme3) and CP2(R6Rme2a), revealing its potential for modulating both lysine and arginine methylation status in non-histone sequences. Structure- and MS-guided derivatization of an initial hit led to proteolytically more stable macrocycles that stabilize the intracellular target. Changes in the global histone H3K9/K36 methylation status were observed in cells treated with CP2.3 for 72 h, although further studies are needed to link this observation directly to KDM4 inhibition. The biological effects of substrate competing cyclic peptides targeting KDM4A may arise both from simple inhibition of KDM4A catalysis, as well as disruption of its binding to chromatin complex; the latter mechanism is likely to be different to the effect of catalytically inactivating small-molecule inhibitors, which compete with the 2OG cosubstrate, but not with histone substrates. We also cannot rule out the possibility that the observed intracellular KDM4A stabilization may in part be due to CP2/CP2.3 proteolytically cleaved fragments, which may, or may not, exert inhibitory effects. Although further studies are necessary to investigate the cell permeability/subcellular distribution, stability and activities of the cyclic peptides, our work highlights the utility of the approach for the generation of potent, substrate competitive and selective KDM inhibitors, and more generally, for developing new approaches to the inhibition of challenging targets.

## Methods

### FIT system

Flexizyme RNA and tRNA^fMet^_CAU_ were prepared as reported^16^. ClAc^L^Tyr-tRNA^fMet^_CAU_ and ClAc^D^Tyr-tRNA^fMet^_CAU_ were synthesized by using the reported flexizyme technology^11^. Translation factors, enzymes and ribosome were prepared and mixed as previously described^27^,^28^,^29^ to reconstitute an *in vitro* translation system used for reprogramming of translation initiation^11^,^17^. The translation reaction mixture contained final concentrations of 50 mM Hepes-KOH (pH 7.6), 100 mM potassium acetate, 2 mM GTP, 2 mM ATP, 1 mM CTP, 1 mM UTP, 20 mM creatine phosphate, 12 mM Mg(OAc)_2_, 2 mM spermidine, 2 mM dithiothreitol, 1.5 ml^−1^ *Escherichia coli* total transfer RNA (Roche), 1.2 μM ribosome, 0.6 μM MTF, 2.7 μM IF1, 0.4 μM IF2, 1.5 μM IF3, 30 μM EF-Tu, 30 μM EF-Ts, 0.26 μM EF-G, 0.25 μM RF2, 0.17 μM RF3, 0.5 μM RRF, 4 μg ml^−1^ creatine kinase, 3 μg ml^−1^ myokinase, 0.1 μM pyrophosphatase, 0.1 μM nucleotide-diphosphatase kinase, 0.1 μM T7 RNA polymerase, 0.73 μM AlaRS, 0.03 μM ArgRS, 0.38 μM AsnRS, 0.13 μM AspRS, 0.02 μM CysRS, 0.06 μM GlnRS, 0.23 μM GluRS, 0.09 μM GlyRS, 0.02 μM HisRS, 0.4 μM IleRS, 0.04 μM LeuRS, 0.11 μM LysRS, 0.03 μM MetRS, 0.68 μM PheRS, 0.16 μM ProRS, 0.04 μM SerRS, 0.09 μM ThrRS, 0.03 μM TrpRS, 0.02 μM TyrRS, 0.02 μM ValRS and 200 μM each proteinogenic amino acids, except for methionine, and 50 μM ClAc^L^Tyr-tRNA^fMet^_CAU_ or ClAc^D^Tyr-tRNA^fMet^_CAU_.

### Preparation of puromycin-fused mRNA library

RNAs consisting of 4−12 repeated NNK random sequences (5′-GGGUU, AACUU UAAGA AGGAG AUAUA CAU AUG (NNK)_*m*_ UGC GGC AGC GGC AGC GGC AGC UAG GACGG GGGGC GGAAA-3′, *m*=4−12) were prepared by *in vitro* transcription according to the reported method^12^. The resulting RNAs were mixed in the following ratio—(NNK)_4_:(NNK)_5_:(NNK)_6_:(NNK)_7_:(NNK)_8_:(NNK)_9_:(NNK)_10_:(NNK)_11_:(NNK)_12_=20^−3^:20^−2^:20^−1^:1:10:10:10:10:10. The mRNA library was ligated with a puromycin linker (5′-CTCCC GCCCC CCGTC C-(SPC18)_5_-CC-puromycin-3′) by T4 RNA ligase. The ligated product was purified by phenol–chloroform extraction and ethanol precipitation.

### *In vitro* selection of cyclic peptides binding to KDM4A

Translation of the first round selection was performed using 156 pmol mRNA-puromycin and 150 μl of translation mixture at 37 °C for 30 min. Subsequently, the translation mixture was incubated at room temperature for 12 min to conjugate the translated peptide with the corresponding mRNA–puromycin. To this solution was added 15 μl of 200 mM EDTA (pH 7.5) then further incubated at 37 °C for 30 min, to proceed peptide macrocyclization. After exchange with the selection buffer (50 mM Tris-HCl (pH 7.6), 150 mM NaCl, 0.05% Tween 20) by gel filtration, 165 μl of blocking solution (100 mM Tris-HCl (pH7.6), 300 mM NaCl, 0.2% acetyl BSA) was added. The resulting peptide library was treated with Dynabeads magnetic beads (His-tag isolation and pulldown, M-280, Invitrogen) at 4 °C for 30 min. The supernatant was then collected, mixed with KDM4A-immobilized magnetic beads (final concentration of KDM4A was adjusted to 1 μM) and incubated at 4 °C for 30 min. After the supernatant was removed, the bead was washed three times with 800 μl of cold selection buffer. To the bead was added 40 μl of room temperature reaction buffer I (50 mM Tris-HCl (pH 8.3), 75 mM KCl, 3 mM MgCl_2_, 10 mM dithiothreitol, 0.5 mM dNTPs, 2 μM CGS3an13.R39 (5′-TTTCC GCCCC CCGTC CTAGC TGCCG CTGCC GCTGC CGCA-3′)) containing 200 units of M-MLV reverse transcriptase (Promega) and 8 units of RNase inhibitor (Promega), and reverse transcribed at 42 °C for 60 min. The collected cDNA was eluted with 800 μl of PCR buffer (10 mM Tris-HCl (pH 7.5), 50 mM KCl, 0.1% Triton X-100, 2.5 mM MgCl_2_, 0.25 mM dNTPs, 0.25 μM T7g10M.F48 (5′-TAATA CGACT CACTA TAGGG TTAAC TTTAA GAAGG AGATA TACAT ATG-3′), 0.25 μM CGS3an13.R39) at 95 °C for 5 min. After addition of Taq DNA polymerase to the eluate, the mixture was used for PCR amplification. The amplified DNA was purified by phenol/chloroform extraction followed by ethanol purification. The resulting DNA library was transcribed *in vitro*, ligated with the puromycin linker and used for the next round of selection.

For the 2nd–6th rounds of selection, the translation reaction (5 μl scale) was carried out (37 °C for 30 min, then room temperature for 12 min). After incubation with 1 μl of 100 mM EDTA (pH 7.5) at 37 °C for 30 min, 3.43 μl of room temperature reaction buffer II (74 mM Tris-HCl (pH 8.3), 44 mM Mg(OAc)_2_, 0.74 mM dNTPs, 29 mM KOH, 4.9 μM CGS3an13.R39, 15 U μl^−1^) M-MLV reverse transcriptase without RNase H activity (Promega)) was added. Reverse transcription of the mRNAs fused on peptides was then performed at 42 °C for 60 min. After addition of 21 μl of selection buffer to the library solution and subsequent buffer exchange with selection buffer by gel filteration, 30 μl of blocking solution was added. The resulting peptide library was applied to free Dynabeads magnetic beads at 4 °C for 30 min and then the supernatant was recovered (negative selection). After repeating the negative selection step 3 times, half of the recovered peptide solution was mixed with KDM4A-immobilized magnetic beads (final concentration of KDM4A was adjusted to 500 nM) and incubated at 4 °C for 30 min. After the supernatant was removed, the beads were washed three times with 60 μl of cooled selection buffer. The collected cDNA–mRNA–peptide fusion was recovered with 100 μl of PCR buffer by incubation at 95 °C for 5 min and amplified by *Taq* DNA polymerase. The amplified DNA was purified by phenol/chloroform extraction followed by ethanol purification. The resulting DNA library was transcribed *in vitro*, ligated with the puromycin linker, then used for the next round of selection. After the fifth and sixth rounds of the selection, the resulting cDNA libraries were cloned into the pGEM-T Easy Vector (Promega) and the individual clones were sequenced.

### Peptides

PolyR (Arg)9 peptide was purchased from AnaSpec (AS-621204). The synthesis of peptides used in this study are as described:

### Chemical synthesis of selected macrocyclic peptides

Amino acids were from CS Bio, Novabiochem, Sigma, TCI, Alfa Aesar and AGTC Bioproducts. Azide-containing amino acids were from Iris Biotech, BCN-conjugates for copper-free click chemistry were purchased from Synaffix (Nijmwegen, The Netherlands), Azides for click chemistry were obtained Baseclick (Tutzing, Germany). All peptides were analysed using an Agilent 1200 series LC-MS system (6120 quadrupole MS) with a Waters Sunfire column. Preparative HPLC purifications were carried out using Shimazu prominence LC-20AP system with a Merck Chromolith Prep column (200–25 mm) or Dionex Ultimate 3,000 system with a Grace Vydac 218TP101522 column.

Method A: peptides were synthesized with an amidated carboxy terminus on a 25 μmol scale using NovaPEG Rink Amide resin (Merck) using standard Fmoc protection chemistry. After coupling of the *N-*terminal L-tyrosine or D-tyrosine residue, their Fmoc group was removed. The resulting N-terminal α-amino group was chloroacetylated by incubating with a solution of 0.5 M chloroacetyl *N*-hydroxysuccinimide ester chloromethylcarbonyloxysuccinimide (ClAc-OSu) in *N*-methylpyrrolidone with rotation for 40 min at room temperature. After washing the resin with dimethylformamide (2 ml, three times) and dichloromethane (2 ml, six times), the peptides were cleaved from the resin and deprotected by incubation with a solution of trifluoroacetic acid (TFA)/1,2-ethanedithiol/triisopropyl silane/water (92.5:2.5:2.5:2.5) with rotation at room temperature for 3 h. The solutions were concentrated *in vacuo* and the cleaved peptides were then precipitated with diethyl ether. The resulting linear peptide pellets were dissolved in 20 ml of water/DMSO (1:1) and triethylamine was added to the solution to give a pH of around 10. After incubation at room temperature for 30 min to effect cyclization, the peptide solution was acidified using TFA then purified by reverse-phase HPLC (Shimazu prominence LC-20AP system with a Merck Chromolith Prep column) in 0.1% aqueous TFA/acetonitrile containing 0.1% TFA gradient. The purified peptides were lyophilized and dissolved in DMSO.

Method B: for the synthesis of cyclic peptides, the linear precursors were prepared by standard solid-phase synthesis using a CS Bio CS336X peptide synthesizer (100 μmol scale) using DIC as coupling reagent. For methylated peptides coupling times were tripled for reaction of the amino acid following the *N*-methylated one.

After cleavage of the N-terminal Fmoc-protecting group a solution of 150 mg of ClAc-OSu in 4 ml dimethylformamide was added to the resin and the mixture was shaken for 3 h. The resin was filtered off and subsequently treated with 4 ml of deprotection solution TFA/triisopropyl silane/water (95:2.5:2.5). After 3 h, the volume was reduced to 1 ml under a nitrogen stream and the peptides were precipitated with cold Et_2_O. The mixture was centrifuged and the supernatant discarded. The solid was taken up in 1.5 ml of triethylammoniumacetate buffer (1 M, pH 8.5) and the pH readjusted to >8 if necessary. In a microwave (Biotage Initiator), the mixture was heated to 80 °C for 10 min and subsequently purified by HPLC (0–45% MeCN in 45 min, 0.1% TFA, Dionex Ultimate 3,000 series, Grace Vydac 218TP101522 column).

### Synthesis of ClAc-OSu

ClAc-OSu was synthesized using a modified literature procedure^30^. A round-bottom flask was charged with 4.00 ml chloroacetylchloride, 6.90 g of K_2_CO_3_ and 80 ml CH_2_Cl_2_, and cooled to 0 °C. *N*-hydroxysuccinimide (5.78 g) was dissolved in 80 ml of MeCN and added slowly to the reaction vessel. The mixture was allowed to warm to room temperature overnight. Subsequently, the slurry was diluted with 500 ml CH_2_Cl_2_, washed with 200 ml HCl (1 M) and the aqueous layer was then counter-extracted with 500 ml CH_2_Cl_2_. The combined organic layers were dried over MgSO_4_, then concentrated to give 3.50 g (36%) of chloromethylcarbonyloxysuccinimide as a colourless solid. ^1^H NMR (400 MHz, CDCl_3_): *δ* (p.p.m.)=4.37 (s, 2 H), 2.85 (s, 4 H). ^13^C NMR (101 MHz, CDCl_3_) *δ* (p.p.m.)=168.4, 163.2, 37.8, 25.5.

### Protein expression and purification of KDM4A

N-terminally Hexa-His-tagged KDM4A (His-KDM4A_1–359_) was produced and purified as described^31^. The KDM4A gene encoding for KDM4A protein (residues 1-359) was cloned into the pNIC-Bio3 plasmid, which incorporates a C-terminal AviTag and expressed with biotin supplement in BL21(DE3)Rosetta co-transfected with pCDF-BirA^32^. His-KDM4A with biotin (His-KDM4A-Bio) was purified using nickel affinity chromatography. Complete (>95%) biotinylation of KDM4A was confirmed using electrospray ionization–MS (Waters Micromass LCT Premier). The enzyme activity was comparable to that of His-KDM4A and the biotin-tag was demonstrated to be accessible to streptavidin binding as determined by AlphaScreen-binding assay^33^. The full-length FLAG-tagged KDM4A_1–1,064_ (ref. 15) was produced in HEK293T cells. HEK293T cells were grown to 80% confluency in DMEM medium (Sigma) supplemented with Glutamax (Life Technologies), 10% fetal bovine serum (FBS, Sigma). FLAG-tagged KDM4A was transfected using polyethyleneimine. Cells were harvested after 48 h, washed with PBS (Sigma) and lysed (45 min, 4 °C) in 50 mM HEPES (pH 7.5), 150 mM NaCl buffer containing protease inhibitor cocktail (Sigma), 10 μM DNase (Sigma) and 0.1% NP40 (Roche). FLAG-tagged KDM4A was purified by immunoprecipitation using anti-FLAG M2 magnetic beads (Sigma) and washed in in 50 mM HEPES (pH 7.5) 150 mM NaCl. The final wash was performed in 50 mM HEPES (pH 7.5) and the resin was used as an enzyme source for the full-length KDM4A.

### Kinetic measurements

IC_50_ values for KDM2–6 were measured using an AlphaScreen assay as described^18^. The appropriate KDM (5 μl) was pre-incubated with an inhibitor (0.1 μl in DMSO) or DMSO for 15 min before the addition of 5 μl mixture of biotinylated peptide substrates, 2OG, ferrous ammonium sulfate (1–10 μM final) and ascorbate (100 μM final) to initiate the demethylation reaction. The reaction was quenched with 5 μl of 30 mM EDTA at predetermined reaction times for each enzyme (initial rate). Demethylated product was detected by addition of 5 μl of AlphaScreen donor and acceptor beads (AlphaScreen General IgG Detection Kit, 0.02 mg ml^−1^ final each) pre-incubated with antibody. The plates were read after 1 h incubation in a BMG Labtech Pherastar FS plate reader. Data were normalized to a no enzyme control and IC_50_ values were calculated using nonlinear regression curve fits using GraphPad Prism. Assay buffer (50 mM HEPES pH 7.5, BSA (0.1% w/v) and Tween-20 (0.01% v/v)) was used throughout, except for dilution of stock ferrous ammonium sulfate solutions where water was used. The enzyme, substrate and antibody concentrations used are described in Supplementary Table 3. A fluorescence-based LSD1 inhibitor screening assay kit (Cayman, 700120) was used for KDM1/LSD1 assay. Assays for prolyl hydroxylase domain 2 and factor inhibiting HIF using matrix-assisted laser desorption/ionization–time of flight MS were performed as previously described^31^. Prism 5.0 (GraphPad) was used to generate and to fit dose–response curves. IC_50_ values were determined by plotting percentage inhibition versus compound concentration relative to positive (vehicle) and negative (no enzyme) controls, using normalized dose–response (variable slope) fit. The reported values are the mean of multiple experiments (*n*≥3). The formaldehyde dehydrogenase coupled KDM assay was used to determine the mode of inhibition^34^, using 0.5 μM His-KDM4A_1–359_. Prism 5.0 (GraphPad) was used to fit in mix-mode inhibition, to determine the kinetic parameters. Binding constants for CPs and H3(1–15)K9me3 were measured using biolayer interferometry (OctetRed 384, ForteBio) in buffer (50 mM HEPES (pH 7.5), 150 mM NaCl, 10 μM ammonium iron(II) sulphate). His-KDM4A_1–359_-Bio (70 nM) was immobilized onto super streptavidin biosensor and subjected to different peptide concentrations in buffer. Data analysis was performed using ForteBio Data Aalysis (v6.4).

### Crystallography

The KDM4A.CP2 complex was prepared by adding CP2 to a final concentration of 0.2 mM in a solution of 0.25 mM (11 mg ml^−1^) His-KDM4A_1–359_ in gel filtration buffer (10 mM HEPES pH 7.5, 500 mM NaCl and 5% glycerol). Crystals were grown by vapour diffusion at 4 °C in 200 nl sitting drops with a 1:1 ratio of sample to well solution (0.1 M propionate-cacodylate-bis-tris propane pH 9.0 and 25% w/v polyethylene glycol 1,500). Crystals of KDM4A.CP2(R6Kme3) complex (0.25 mM His-KDM4A_1–359_, 1 mM NiCl_2_, 2.5 mM NOG and 5 mM CP2(R6Kme3) were grown by vapour diffusion at 4 °C in 300 nl sitting drops with a 1:2 ratio of sample to well solution (0.1 M bicine pH 9.0 and 10% w/v polyethylene glycol 6,000). Crystals were cryo-protected by transferring to a solution of mother liquor supplemented with 25% v/v glycerol before being flash frozen in liquid N_2_. An additional 0.5 mM CP2 was added to the cryoprotectant for KDM4A.CP2 crystals.

Data were collected from single crystals at 100 K at the Diamond Light Source, beamline I04-1. Data for the KDM4A.CP2 complex were processed using XDS^35^ and SCALA^36^; data for the CP2(R6Kme3) complex were processed using HKL2000 (ref. 37). The structures were solved by molecular replacement using PHASER^38^ and PDB 2OX0 as the initial model. The majority of the KDM4A.CP2 structure refinement was carried out with Phenix^39^, with iterative rebuilding of the model using COOT^40^. Final rounds of slowcool-simulated annealing refinement using the maximum-likelihood function and bulk-solvent modelling in CNS^41^ proceeded until the decreasing *R*/*R*_free_ no longer converged. KDM4A.CP2(R6Kme3) complex was refined by alternative cycles of CNS^41^ and PHENIX^39^ using the ML function and bulk-solvent modelling. All residues were in acceptable regions of the Ramachandran plot as calculated by PROCHECK^42^ and/or MOLPROBITY^43^. Data collection and refinement statistics are in Supplementary Table 2.

### Cell culture

HEK293T, HeLa and U2OS cells were obtained from the ATCC (LGC Standards, UK). FBS, L-glutamine and DMEM medium were purchased from Sigma; PBS and OptiMEM were from ThermoFisher Scientific. All cell lines were cultured in DMEM supplemented with 10% FBS, 2 mM L-glutamine and 1 × Pen-Strep at 37 °C and 5% CO_2_ unless otherwise stated.

### MS-based peptide degradation assay

HEK 293T cells (approximately 1 × 10^7^ cells) in a 75 cm^2^ flask were washed with PBS, harvested with trypsin, quenched with 10% FBS (Sigma)-containing DMEM (Sigma) and centrifuged at 200 *g* for 3 min at 4 °C. The pelleted cells were re-suspended in 1 ml buffer containing 20 mM Tris-HCl (pH 7.6) and 140 mM NaCl. The suspension was freeze–thawed five times using liquid nitrogen and a heating block set at 25 °C, vortexing briefly between each thawing, followed by centrifugation (14,000 *g*, 10 min, 4 °C).

CP2(T13Z) was added to 250 μl lysate, yielding a final concentration of 200 μM peptide. The contents were incubated at 37 °C and 30 μl aliquots withdrawn at various time points. Analyses were performed using a Thermo Exactive mass spectrometer equipped with Waters Acquity liquid chromatography system. Instrument control and data processing were performed using Thermo Xcalibur Software. The system was calibrated on the day of the analysis. The mass accuracy with external calibration (as used for these experiments) was better than 5ppm for 24 hours following calibration. Electrospray source conditions were adjusted to maximize sensitivity. Each sample (1 μl) was injected into the system with a 2.1 × 50 mm (3 μm) Purospher STAR RP-18 endcapped column at a flow rate of 0.4 ml min^−1^. The oven was held at 40 °C throughout the analysis. Solvent A was aqueous 0.1% formic acid and solvent B was acetonitrile. Peptides were eluted from the column using a gradient of 5–100% solvent B over 8 min. All raw data files were analysed with MestReNova V.10.

### Cell thermal stability assays

For CETSA melting curve experiments, 80% confluent U2OS cells in a 75 cm^2^ flask were transfected with a plasmid encoding full-length Flag-tagged KDM4A_1–1,064_ (ref. 15) or KDM4E_1–506_ (ref. 20) using Lipofectamine 2000 (Thermo Scientific) and incubated for 4 h. The cells were washed with PBS buffer and media replaced with fresh DMEM with 10% FBS, supplemented with cyclic peptides in DMSO (0.1% DMSO final). Cell dosing was carried out for 20 h at 37 °C. Control cells were dosed only with DMSO. The cells were trypsinized and washed thrice with PBS, by centrifugation (300 *g*, 3 min, 4 °C). Cells were re-suspended in PBS supplemented with Complete Protease inhibitor cocktail (Sigma) and divided into 8–10 aliquots (100 μl) containing equal cell numbers in PCR tubes. For ITDRF CETSA experiments, approximately 6 × 10^6^ cells were seeded evenly into nine 25 cm^2^ flask and adhered before transfection. After 4 h of incubation, the cells were washed with PBS buffer and the media replaced with fresh DMEM with 10% FBS supplemented with cyclic peptides at a final concentration between 0 and 4,000 nM (0.1% DMSO final). Cells were trypsinized and washed thrice with PBS using centrifugation (300 *g*, 3 min, 4 °C). Cells were re-suspended in PBS supplemented with protease inhibitor cocktail (Sigma), aliquots (100 μl) containing equal cell numbers in PCR tubes were prepared. For CETSA melting curve experiments, each PCR tube was incubated for 3 min in a heating block at different temperatures, then at room temperature for 3 min. For ITDRF, tubes were incubated at approximately the *T*_m_ of the proteins of interest as determined by CETSA melting curve experiments, for example, 55 °C (*T*_m_ for actin) for 3 min, followed by room temperature for 3 min. The tubes were centrifuged (300 *g*, 3 min, 4 °C). The supernatant was removed and cells suspended in lysis buffer (100 mM HEPES, 300 mM NaCl, 2% NP-40 and 10 mM EDTA, pH 7.4), supplemented with protease inhibitor cocktail. The tubes were incubated at 4 °C for 1 h, with vortexing every 20 min. Samples were centrifuged for 30 min at >16,000 *g* at 4 °C to pellet cell debris and precipitated proteins.

The samples (supernatant) were run on SDS–PAGE (NuPage Novex Bis-Tris 4–12% polyacrylamide gels with NuPAGE MES SDS running buffer (Thermo Scientific)) and transferred onto nitrocellulose membranes using either the iBlot blotting system. FLAG-KDM4A levels were determined by immunoblotting using a monoclonal mouse anti-FLAG primary antibody (F1804, Sigma), at a dilution of 1: 1,000 in PBS-T buffer containing Seablock Reagent Salmon Plasma (2% v/v, Calbiochem). An additional chicken anti-β-actin primary antibody (ab13822, Abcam) was also used. A goat anti-mouse fluorescent secondary antibody (800 nm, 1:10,000, Thermo Scientific) and anti-chicken fluorescent secondary antibody (680 nm, 1:10,000, Thermo Scientific) were used for imaging on a LI-COR Odyssey CLx imaging system. Western blotting band intensities were quantified using Image Studio Ver. 3.1 (LI-COR Biosciences) and plotted in Prism 6.07 software (GraphPad Software Inc.) to generate thermal stability curves.

### Data availability

The crystal structures KDM4A.Ni(II).CP2 and KDN4A.Ni(II).CP2(R6Kme3) have been deposited under PDB accession codes 5LY1 and 5LY2, respectively. All other data are available from the authors upon reasonable request.

## Additional information

**How to cite this article:** Kawamura, A. *et al*. Highly selective inhibition of histone demethylases by *de novo* macrocyclic peptides. *Nat. Commun.* **8**, 14773 doi: 10.1038/ncomms14773 (2017).

**Publisher's note:** Springer Nature remains neutral with regard to jurisdictional claims in published maps and institutional affiliations.

## Supplementary Material

Supplementary Information Supplementary figures, supplementary tables, supplementary methods and supplementary references.

## Figures and Tables

**Table 1:** Potency and selectivity of cyclic peptide hits from the RaPID system.

	**IC**_**50**_ **(nM)**				
	^**D**^**Tyr-Library**		^**L**^**Tyr-Library**		
	**CP1**	**CP2**	**CP3**	**CP4**	**CP5**
KDM4A	>10^5^	42	>10^5^	20	313
KDM4B	—	33	—	6	472
KDM4C	—	39	>10^5^	17	123
KDM4D	—	6,270	—	6,260	>10^4^
KDM4E	—	9,200	—	4,700	8,900
KDM2A	—	>10^4^	—	>10^4^	>10^4^
KDM3A	—	>10^4^	—	9,900	>10^4^
KDM5C	—	>10^4^	—	>10^4^	>10^4^
KDM6B	—	6,800	—	7,200	>10^4^
KDM1A	—	>10^4^	—	>10^4^	>10^4^
PHD2	—	>10^6^	—	>10^6^	>10^6^
FIH	—	>10^6^	—	>10^6^	>10^6^
					
KDM4A Binding					
*K*_d_ (nM)		29.8		36.0	173
*k*_on_ (1/Ms)		1.37 × 10^5^		2.18 × 10^4^	6.2 × 10^4^
*k*_diss_ (1/s)		4.07 × 10^−3^		7.84 × 10^−4^	1.07 × 10^−2^

2OG, 2-oxoglutarate; FIH, factor inhibiting HIF; IC_50_, half-maximal inhibitory concentration; KDM, histone demethylase; LSD, lysine-specific demethylase; MALDI–TOF, matrix-assisted laser desorption/ionization–time of flight; MS, mass spectrometry; PHD2, prolyl hydroxylase domain 2; RaPID, Random nonstandard Peptides Integrated Discovery.

KDM IC_50_ values were determined using AlphaScreen, except for KDM1A/LSD1 where a fluorescence-based assay was used. MALDI–TOF MS assays were used for counterscreening against other 2OG oxygenases. Binding constants for KDM4A were measured using biolayer interferometry.

**Table 2:** Structure activity relationships for CP2 analogues.

		**IC**_**50**_ **(nM)**				**IC**_**50**_ **(nM)**	
		**KDM4A**	**KDM4C**			**KDM4A**	**KDM4C**
1	CP2	42	29	12	T13Z	110	60
2	Linear CP2	172	144	13	C14meC	9	23
3	^D^Y1^L^Y	66	78	14	T13meT	48	53
4	R6A	2,700	3,900	15	V2meV	37	60
5	R6F	5,500	>10^4^	16	^D^Y1me^D^Y	264	192
6	R6AcK	2,400	5,900	17	G8^D^A	10	29
7	R6Cit	283	695	18	G8^D^A/Y12^4F^F	10	9
8	R6K	24	112	19	CP2.1	27	15
9	R6Kme3	12	—	20	CP2.2	100	274
11	CP2(polyR)	1.8	0.8	21	CP2.3	110	69
				22	CP2.3(R6A)	—	>10^4^

IC_50_, half-maximal inhibitory concentration.

IC_50_ values were determined using the AlphaScreen method. All peptides were cyclic, except for Linear CP2. Peptides are named as derivatives of CP2 (original, residue number, modified residue). CP2.1, CP2.2 and CP2.3 contained multiple modifications (see Supplementary Fig. S6): CP2.1—G8DA/C14meC, CP2.2—V2meV/G8DA/T13meT/C14meC, CP2.3—V2meV/G8DA/Y12^4F^F/T13P/C14meC; (meX: N methylation of X, ^D^X: D-amino acid). IC_50_ value of polyR alone was 40 nM against KDM4A.
