# Supplementary material for: Highly selective inhibition of histone demethylases by de novo macrocyclic peptides
Source: Nat Commun. 2017 Apr 6;8:14773. doi: 10.1038/ncomms14773 (PMC5384220; doi:10.1038/ncomms14773)
Supplement: Supplementary Information — Supplementary figures, supplementary tables, supplementary methods and supplementary references. [file ncomms14773-s1.pdf]

**a**

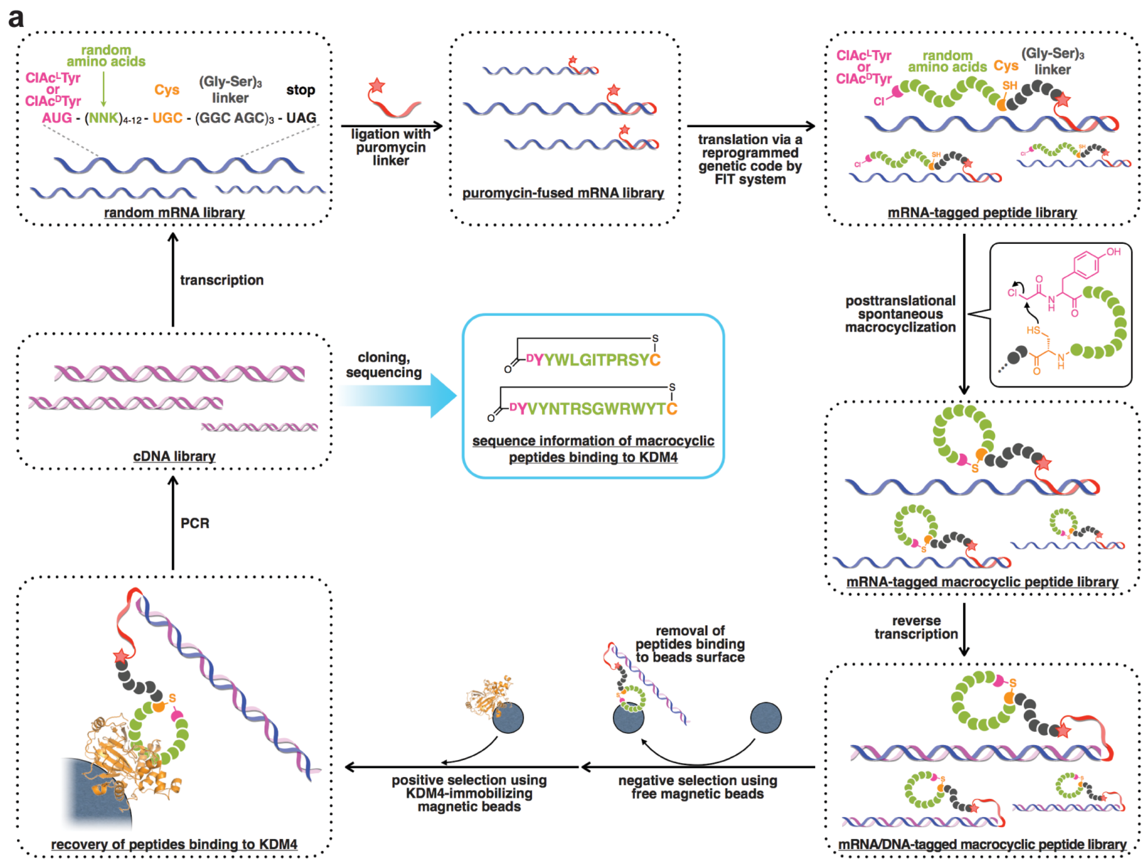


**b**

| peptide name | peptide sequence | number of clones (5th round) | number of clones (6th round) |
|---|---|---|---|
| CP2 | $^{D}$YVVNTRSGWRWYTC | 2 | 7 |
| CP1 | $^{D}$YYWLGITPRSYC | 2 | 0 |
| CP6 | $^{D}$YVRTRRSGLIFFRC | 1 | 0 |
| CP7 | $^{D}$YSRYKQSGILFC | 1 | 0 |
| CP8 | $^{D}$YLRRKCLSVKPKSC | 1 | 0 |
| CP9 | $^{D}$YFRRKCARLPRSKC | 0 | 1 |
| CP10 | $^{D}$YLSHYCLLWCC | 0 | 1 |

**c**

| peptide name | peptide sequence | number of clones (5th round) | number of clones (6th round) |
|---|---|---|---|
| CP3 | $^{L}$YWFYTPAIRWC | 4 | 3 |
| CP4 | $^{L}$YLKSRRSGLTWIPC | 0 | 4 |
| CP5 | $^{L}$YSKYTQSGVRWVC | 1 | 3 |
| CP11 | $^{L}$YYYILNYHDC | 2 | 0 |
| CP12 | $^{L}$YECRSFFTNSGC | 1 | 0 |

**Supplementary Figure 1.** ***In vitro*** **selection of cyclic peptides binding to KDM4A by RaPID selection.** (a) An overview of the RaPID selection process.[1] Identified sequenced peptide “hits” from (b) $^{D}$Tyr- and (c) $^{L}$Tyr-libraries.

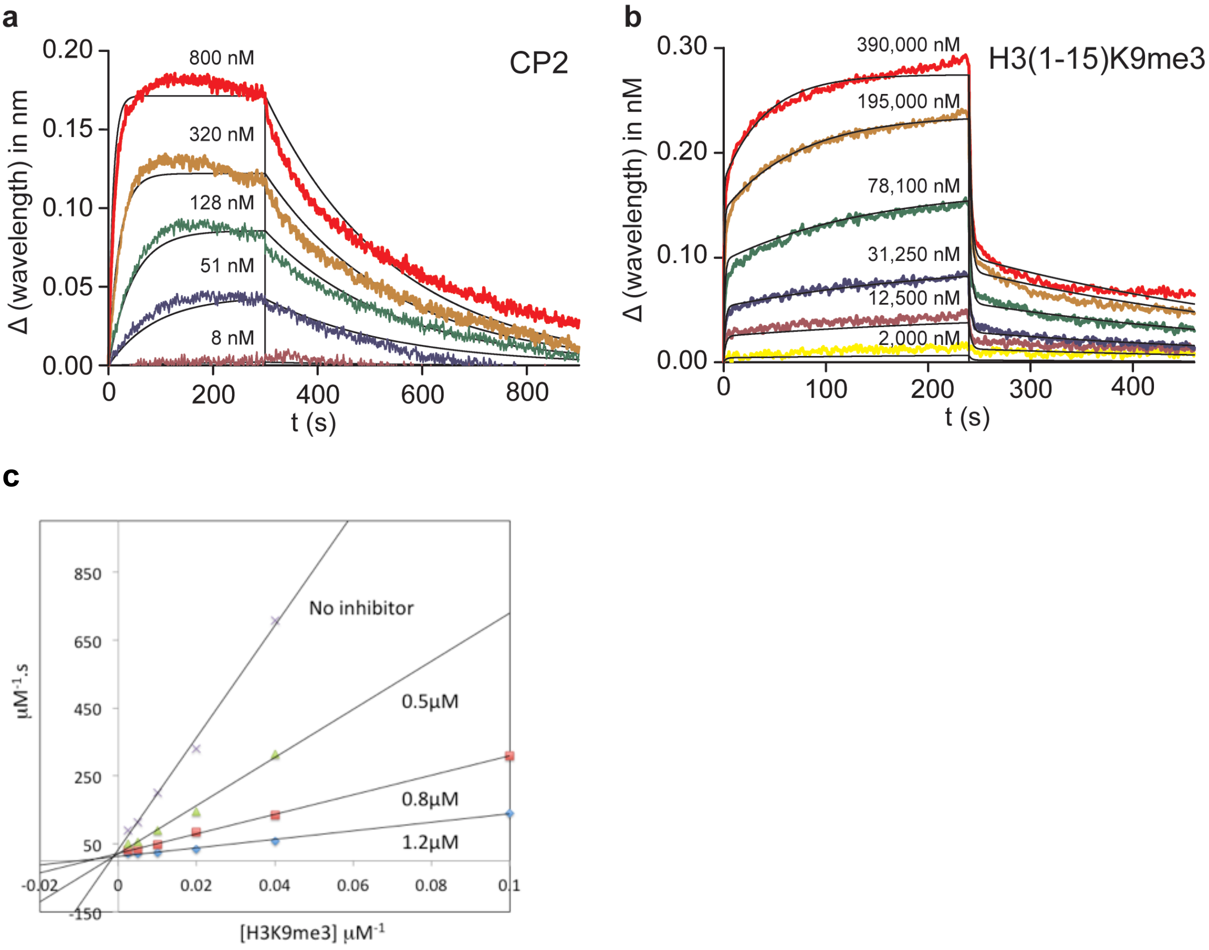


**Supplementary Figure 2. Kinetic analyses of CP2 binding with KDM4A$_{1\text{-}359}$.**
(a, b) Analysis of CP2 and histone H3K9me3 binding to KDM4A by biolayer inteferometry (BLI). The overlay of binding curves of different concentrations of (a) CP2 and (b) H3(1-15)K9me3 with KDM4A. Biotinylated KDM4A$_{1\text{-}359}$ was immobilized and analysed for binding to peptides at different concentrations. $K_d$ (H3(1-15)K9me3) = 110 ± 7.5μM. (c) CP2 competes with H3K9me3 peptide. KDM4A activity on H3(1-15)KMe3 demethylation was measured in the presence of varying concentrations of CP2 using a FDH assay as described.[2] The concentration of KDM4A in the assay mixture was 0.5 μM. Kinetic parameters were calculated using GraphPad Prism. $K_i$ = 0.18 μM, α = 4.73.

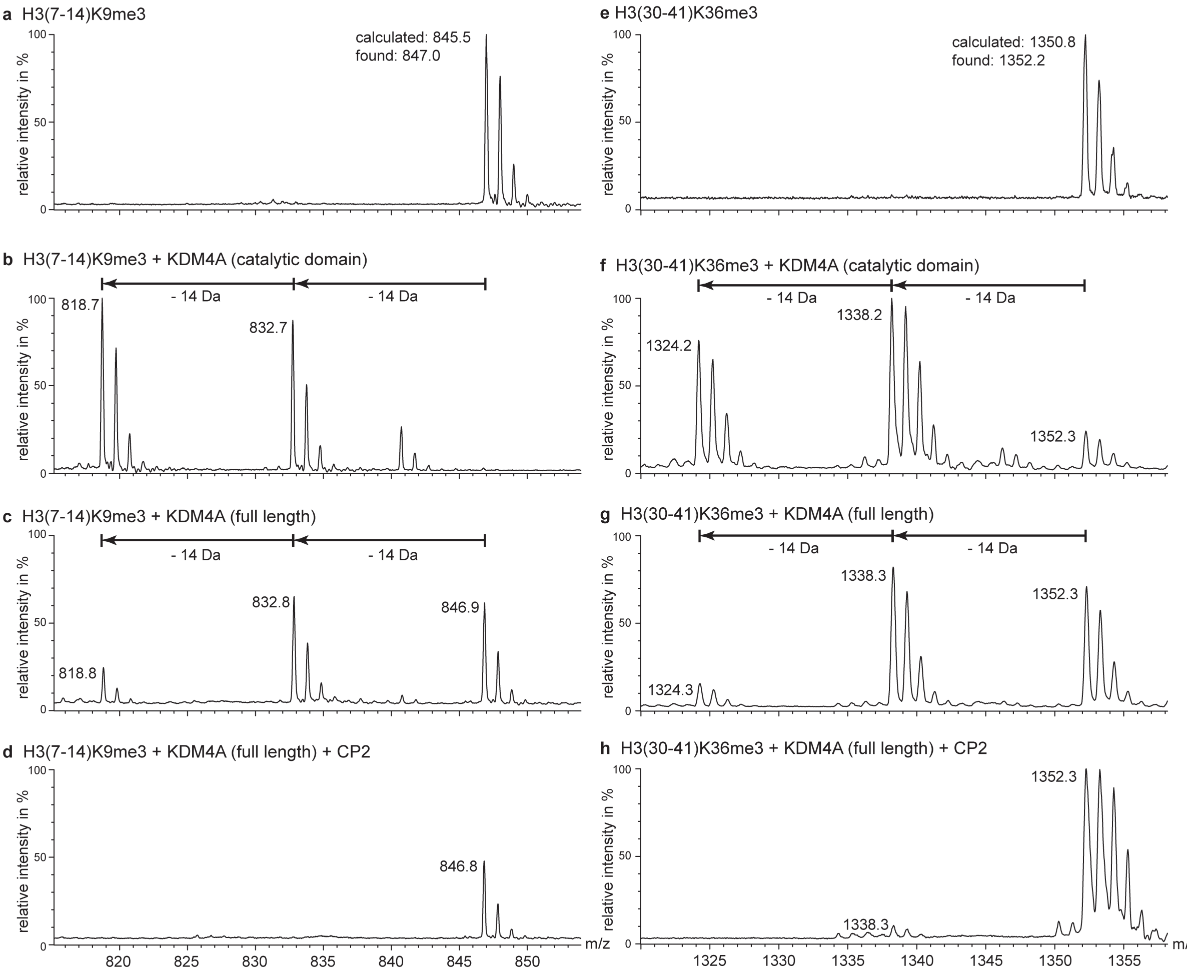


**Supplementary Figure 3. CP2 inhibits the demethylation of H3K9me3 and H3K36me3 by full length KDM4A *in vitro*.** (a)-(h) Histone H3(7-14)K9me3 and H3(30-41)K36me3 peptides (10 µM) were incubated with both the full length $KDM4A_{1\text{-}1064}$ and catalytic domain only $KDM4A_{1\text{-}359}$, with or without CP2 (10 µM) in an assay mixture containing Fe(II) (10 µM), 2OG (100 µM) and sodium ascorbate (100 µM) in 50 mM HEPES (pH 7.5) at 37°C for 1 h. The assay products were analysed by MALDI-TOF MS. FLAG-tagged full length $KDM4A_{1\text{-}1064}$ was transiently expressed in HEK293T cells and purified by FLAG-tag immunoprecipitation as previously described.[3]

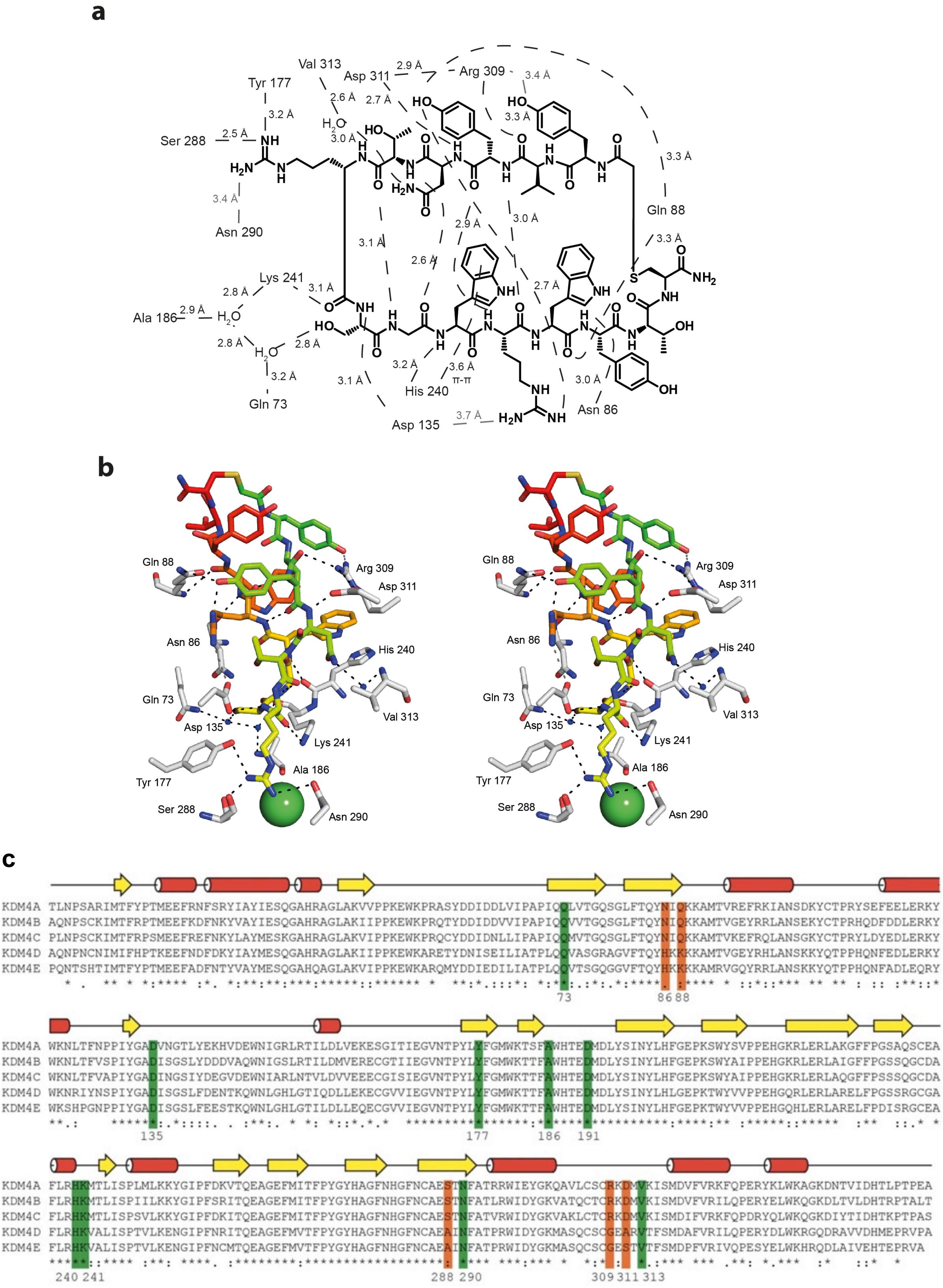


**Supplementary Figure 4. Interactions between the cyclic peptide CP2 and KDM4A.** (**a**) 2D view of polar interactions between CP2 and KDM4A. (**b**) Stereoview (wall eyed) of CP2 interactions (**c**) Sequence alignment of KDM4A-E. Residues interacting with CP2 are highlighted. Green indicates interacting residues that are conserved in all enzymes, orange indicates interacting residues that differ between KDM4A-C and KDM4D/E, which are likely responsible for the intra-subfamily selectivity of CP2.

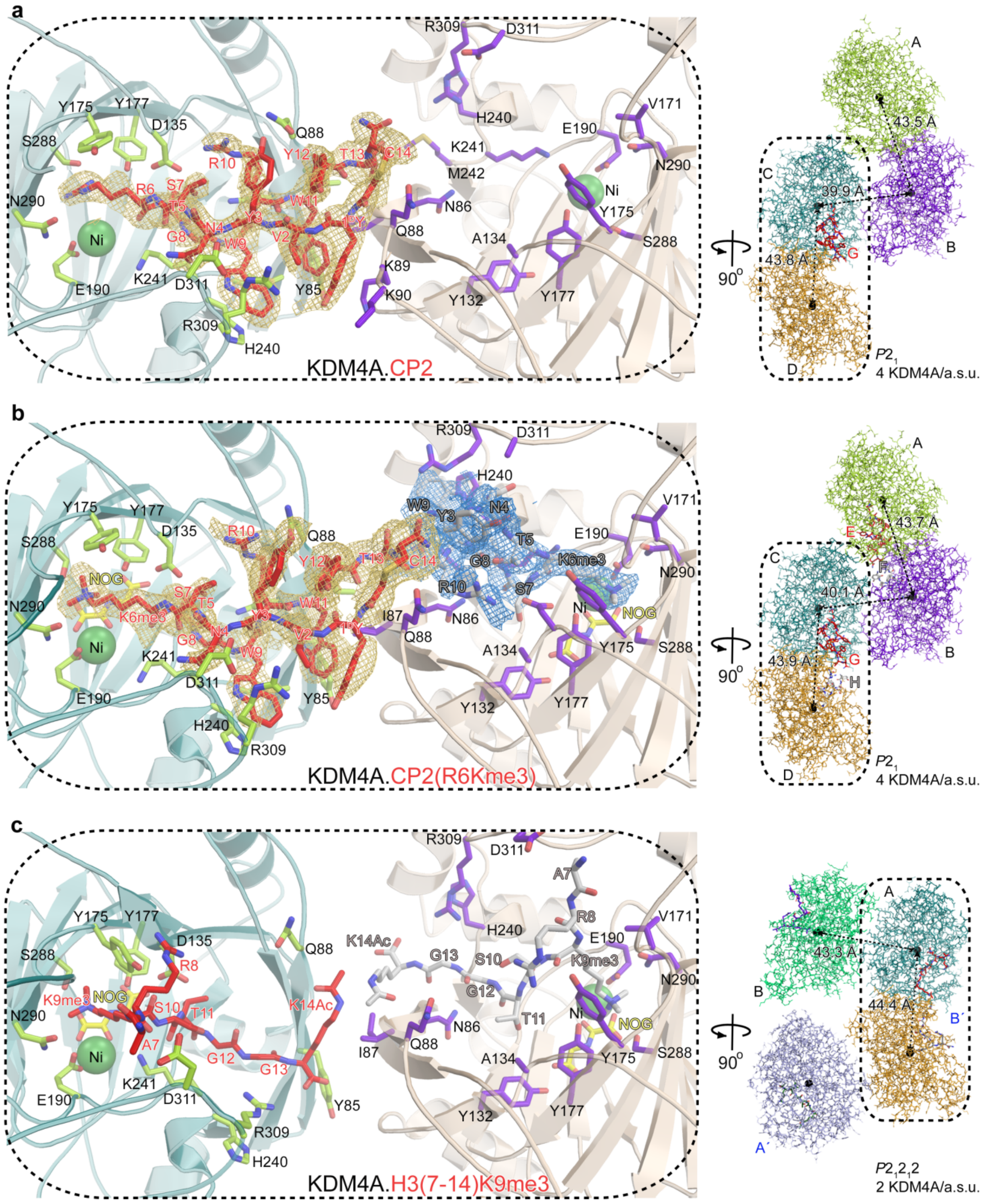


**Supplementary Figure 5. Comparison of KDM4A structures in complex with (a) CP2, (b) CP2(R6Kme3) and (c) H3(7-14)K9me3 (PDB: 2OQ6), showing different electron densities (*F*o-*F*c OMIT) for the cyclic peptides contoured to 3σ.** Both the KDM4A.CP2 and KDM4A.NOG.CP2(R6Kme3) complexes crystallise in the $P2_1$ space group with 4 KDM4A molecules per asymmetric unit (a.s.u)(chains A-D, right panels in **a** and **b**). However, in contrast to KDM4A.CP2 (a), KDM4A.CP2(R6Kme3)(b) complex has two copies of the cyclic peptide bound (peptides denoted as chains E and G (red), bound to KDM4A chains A (green) and C (cyan), respectively in (b)). In addition, electron density was observed for partial occupancy of CP2(R6Kme3) (Tyr3-Arg10 in chains F and H) in the active site of the remaining KDM4A molecules (in chains B (purple) and D (gold) in (b)). The rest of the CP2(R6Kme3) peptides in chains F and H appear to be disordered due to steric clashes with the neighbouring CP2(R6Kme3) molecules in chains E and G. (**c**) A′ and B′ are $P2_12_12$-symmetry related molecules. Dotted lines represent distances between metal centres. From the *N*-terminus, both CP2 and CP2(R6Kme3) make multiple (mostly similar) interactions with KDM4A β5 (aa 85, 86, 87 and 88), β6 (aa 135), DSBH βI (aa 175 and 177), a loop between DSBH strands βIV and βV (aa 240-242), DSBH βVIII (288 and 290) and a *C*-terminal region (aa 309 and 311). The combined structures reveal 'induced fit' nature of cyclic peptide binding to KDM4A residues (a,b) relative to that of histone substrate structure (c) including Tyr175, Lys241, Arg309 and Asp311. Superimposition of the CP2 and CP2(R6Kme3) peptides (**Fig. 3a**) reveals similar side chain conformations of CP residues except for Tyr3, Asn4 and Arg10.

**a**

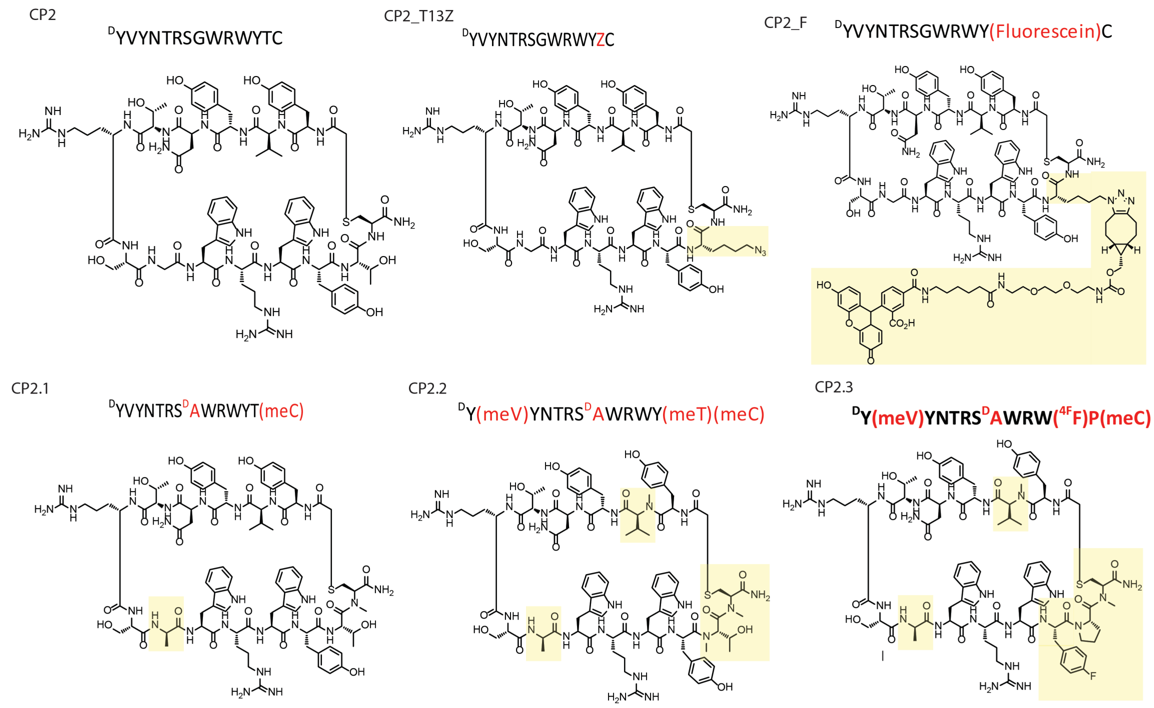


**b**

| Peptides | Sequence | Formula | | m/z calculated | m/z found |
|---|---|---|---|---|---|
| 1. CP2 | $^{D}$YVYNTRSGWRWYTC | $C_{88}H_{118}N_{24}O_{22}S^{2+}$ | $[M+2H^{+}]^{2+}$ | 947.4281 | 947.4302 |
| 2. Linear CP2 | $^{D}$YVYNTRSGWRWYTC | $C_{86}H_{118}N_{24}O_{21}S^{2+}$ | $[M+2H^{+}]^{2+}$ | 927.4307 | 927.4351 |
| 3. CP2( $^{D}$Y1$^{L}$Y) | $^{L}$YVYNTRSGWRWYTC | $C_{88}H_{117}N_{24}O_{22}SNa^{2+}$ | $[M+Na^{+}+H^{+}]^{2+}$ | 958.4191 | 958.4148 |
| 4. CP2(R6A) | $^{D}$YVYNTASGWRWYTC | $C_{85}H_{110}N_{21}O_{22}SNa^{2+}$ | $[M+Na^{+}+H^{+}]^{2+}$ | 915.8871 | 915.8906 |
| 5. CP2(R6F) | $^{D}$YVYNTFSGWRWYZC | $C_{93}H_{117}N_{24}O_{21}S^{2+}$ | $[M+2H^{+}]^{2+}$ | 980.4218 | 980.4169 |
| 6. CP2(R6AcK) | $^{D}$YVYNT(KAc)SGWRWYZC | $C_{92}H_{122}N_{25}O_{22}SNa^{2+}$ | $[M+Na^{+}+H^{+}]^{2+}$ | 991.9402 | 991.9354 |
| 7. CP2(R6Cit) | $^{D}$YVYNT(Cit)SGWRWYZC | $C_{90}H_{118}N_{26}O_{22}SNa_{2}^{2+}$ | $[M+2Na^{+}]^{2+}$ | 995.4210 | 995.4172 |
| 8. CP2(R6K) | $^{D}$YVYNTKSGWRWYZC | $C_{90}H_{119}N_{25}O_{21}SNa_{2}^{2+}$ | $[M+2Na^{+}]^{2}$ | 981.9259 | 981.9255 |
| 9. CP2(R6Kme3) | $^{D}$YVYNT(Kme3)SGWRWYTC | $C_{91}H_{123}N_{22}O_{22}S^{+}$ | $[M+H^{+}]^{+}$ | 991.9584 | 991.9569 |
| 10. CP2(R6me2a) | $^{L}$YVYNT(Rme2a)SGWRWYTC | $C_{90}H_{122}N_{24}O_{22}S^{+}$ | $[M+2H]^{2+}$ | 961.4438 | 961.4451 |
| 11.CP2(polyR) | $^{D}$YVYNTRSGWRWYTC-BRRRRRRRRR | $C_{145}H_{232}O_{32}N_{61}S^{3+}$ | $[M+3H]^{3+}$ | 1123.9639 | 1123.9439 |
| 12. CP2(T13Z) | $^{D}$YVYNTRSGWRWYZC | $C_{90}H_{119}N_{27}O_{21}S^{2+}$ | $[M+2H^{+}]^{2+}$ | 995.9289 | 995.9254 |
| 13. CP2(C14meC) | $^{D}$YVYNTRSGWRWYT(meC) | $C_{89}H_{120}N_{24}O_{22}S^{+}$ | $[M+2H^{+}]^{2+}$ | 954.4359 | 954.4373 |
| 14. CP2(T13meT) | $^{D}$YVYNTRSGWRWY(meT)C | $C_{89}H_{120}N_{24}O_{22}S^{2+}$ | $[M+2H^{+}]^{2+}$ | 954.4359 | 954.4376 |
| 15. CP2(V2meV) | $^{D}$Y(meV)YNTRSGWRWYTC | $C_{89}H_{120}N_{24}O_{22}S^{2+}$ | $[M+2H^{+}]^{2+}$ | 954.4359 | 954.4360 |
| 16. CP2($^{D}$Y1me$^{D}$Y) | (me$^{D}$Y)VYNTRSGWRWYTC | $C_{89}H_{120}N_{24}O_{22}S^{2+}$ | $[M+2H^{+}]^{2+}$ | 954.4359 | 954.4348 |
| 17. CP2(G8$^{D}$A) | $^{D}$YVYNTRS$^{D}$AWRWYTC | $C_{89}H_{120}N_{24}O_{22}S^{2+}$ | $[M+2H^{+}]^{2+}$ | 954.4359 | 954.4344 |
| 18. CP2(G8$^{D}$A/Y12$^{4F}$F) | $^{D}$YVYNTRS$^{D}$AWR($^{4F}$F)Y(Z)C | $C_{91}H_{122}FN_{27}O_{20}S^{2+}$ | $[M+2H^{+}]^{2+}$ | 981.9527 | 981.9510 |
| 19. CP2.1 | $^{D}$YVYNTRS$^{D}$AWRWYT(meC) | $C_{90}H_{122}N_{24}O_{22}S^{2+}$ | $[M+2H^{+}]^{2+}$ | 961.4438 | 961.4419 |
| 20. CP2.2 | $^{D}$Y(meV)YNTRS$^{D}$AWRWY(meT)(meC) | $C_{92}H_{126}N_{24}O_{22}S^{2+}$ | $[M+2H^{+}]^{2+}$ | 975.4594 | 975.4621 |
| 21. CP2.3 | $^{D}$Y(meV)YNTRS$^{D}$AWRW($^{4F}$F)P(meC) | $C_{92}H_{123}FN_{24}O_{20}S^{2+}$ | $[M+2H^{+}]^{2+}$ | 967.4520 | 967.4483 |
| 23. CP4 | $^{L}$YLKSRRSGLTWIPC | $C_{78}H_{125}N_{23}O_{19}S^{2+}$ | $[M+2H+]^{2+}$ | 859.9616 | 859.9627 |
| 24. CP5 | $^{L}$YSKYTQSGVRWVC | $C_{73}H_{107}N_{20}O_{20}S^{2+}$ | $[M+H+]^{+}$ | 1615.7686 | 1615.7620 |
| 25. CP2(Fl) | $^{D}$YVYNTRSGWRWY(Fluorescein)C | $C_{134}H_{170}N_{30}O_{32}S^{2+}$ | $[M+2H^{+}]^{2+}$ | 1371.8154 | 1371.8123 |

**Supplementary Figure 6. Structures and MS analysis of the macrocyclic peptide hits and derivatives.** (**a**) Structures of cyclic CP2 and its derivatives used in in vitro and cell based assays. (**b**) HRMS of peptides used in this study. All peptides are cyclic (thioether bond between the *N*- and *C*-terminal amino acids) except for entry 2. Abbreviations: KAc – $N^{\varepsilon}$-acetylated lysine, Cit – citrulline, Kme3 – $N^{\varepsilon}$-trimethylated lysine, me2a – asymmetric dimethylation, B –β-Ala; meX – $N^{\alpha}$-methylated X, $^{4F}$F – 4-fluorophenylalanine, Z – *L*-azidolysine, Fl – fluorescein attached via a cycloaddition 'click' reaction. All modified residues are highlighted in red. For peptide 22 (CP2.3(R6A): $^{D}$Y(meV)YNTAS$^{D}$AWRW($^{4F}$F)P(meC)), HRMS could not be obtained. Calculated mass 1847.8; observed mass was 1850 (± 2 Da) using MALDI-TOF MS.

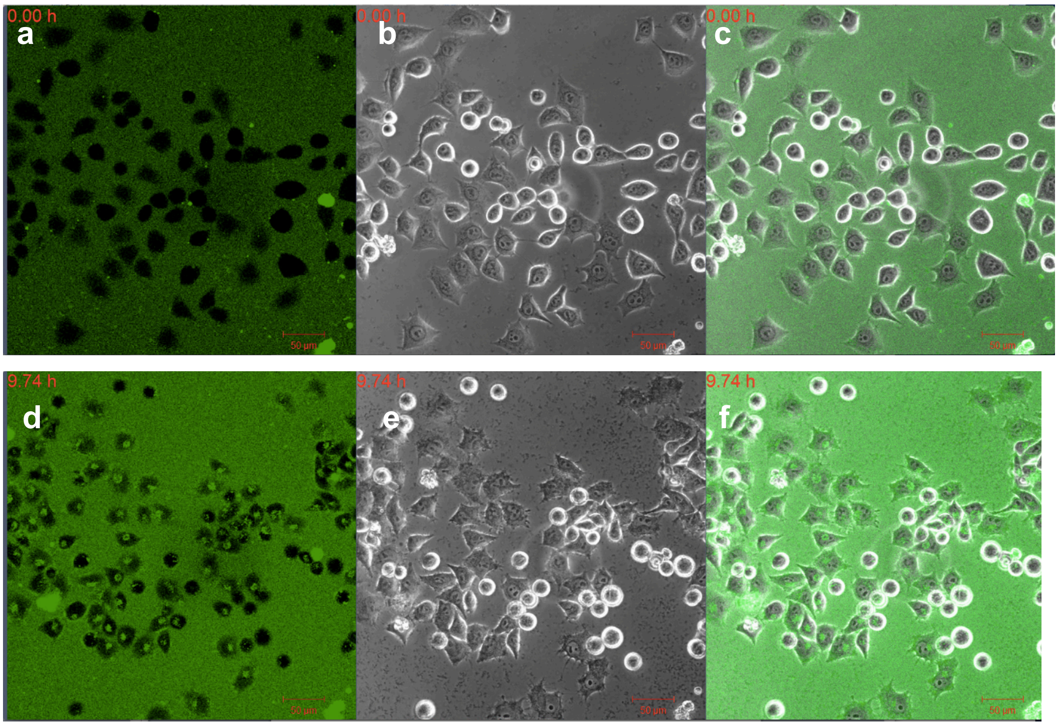


**Supplementary Figure 7. Time-lapse confocal imaging of HeLa cells incubated with fluorescein-conjugated CP2 (CP2(Fl)).** HeLa cells were treated with 10 μM CP2(Fl) and imaged over a 10 h period. Panels (**a-c**) are time zero images (fluorescence, bright-field and merged images, respectively) and panels (**d-f**) are images taken after 10 h incubation. Bar represents 50 μm. Note that we have found that the cell-permeability of (fluorescently) tagged and untagged cyclic peptides can vary (unpublished results).

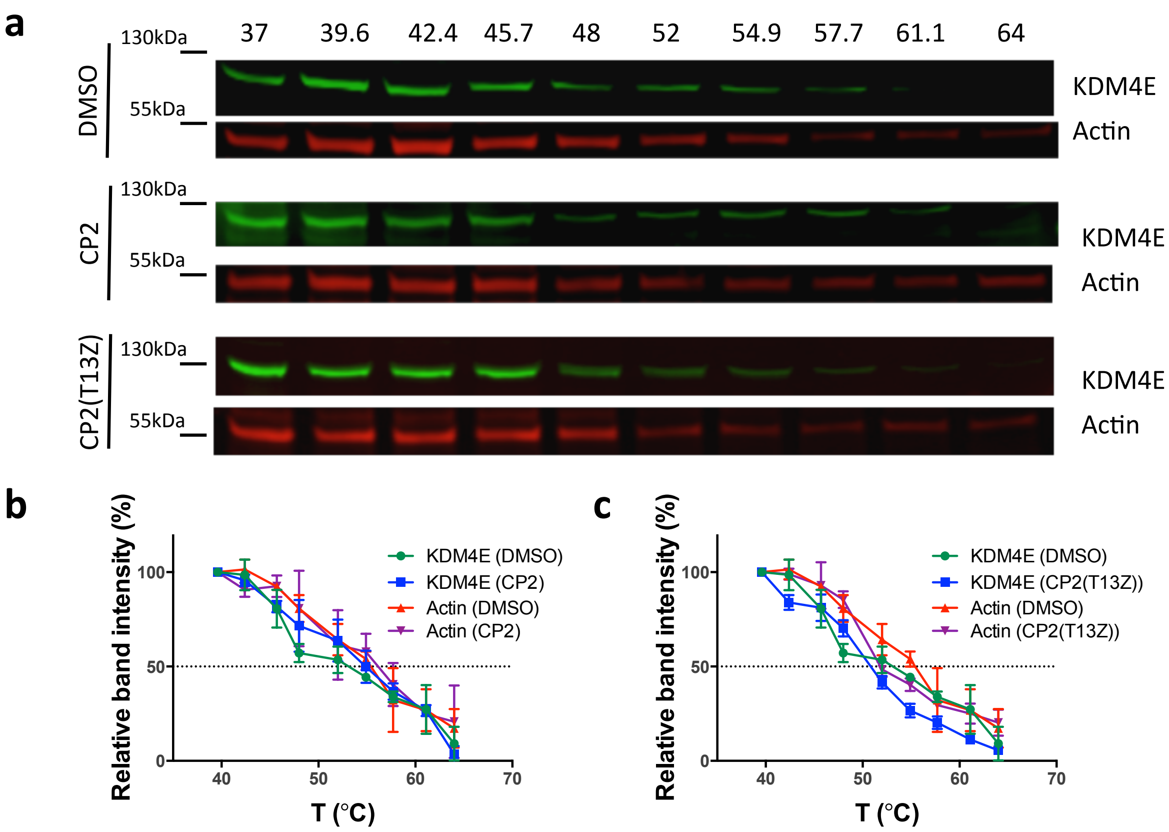


**Supplementary Figure 8. CP2 peptides do not stabilize KDM4E in cells**. Western blots (a) and CETSA melting curves (b, c) for overexpressed Flag-tagged KDM4E in U2OS with and without cyclic peptide (CP2, CP2(T13Z)) treatment (0.5 µM). No significant changes in the Tm shift are observed for both KDM4E and actin upon peptide treatment. Representative western blot figures are shown. Avg +/- std dev (n = 2) are plotted for the melting curves.

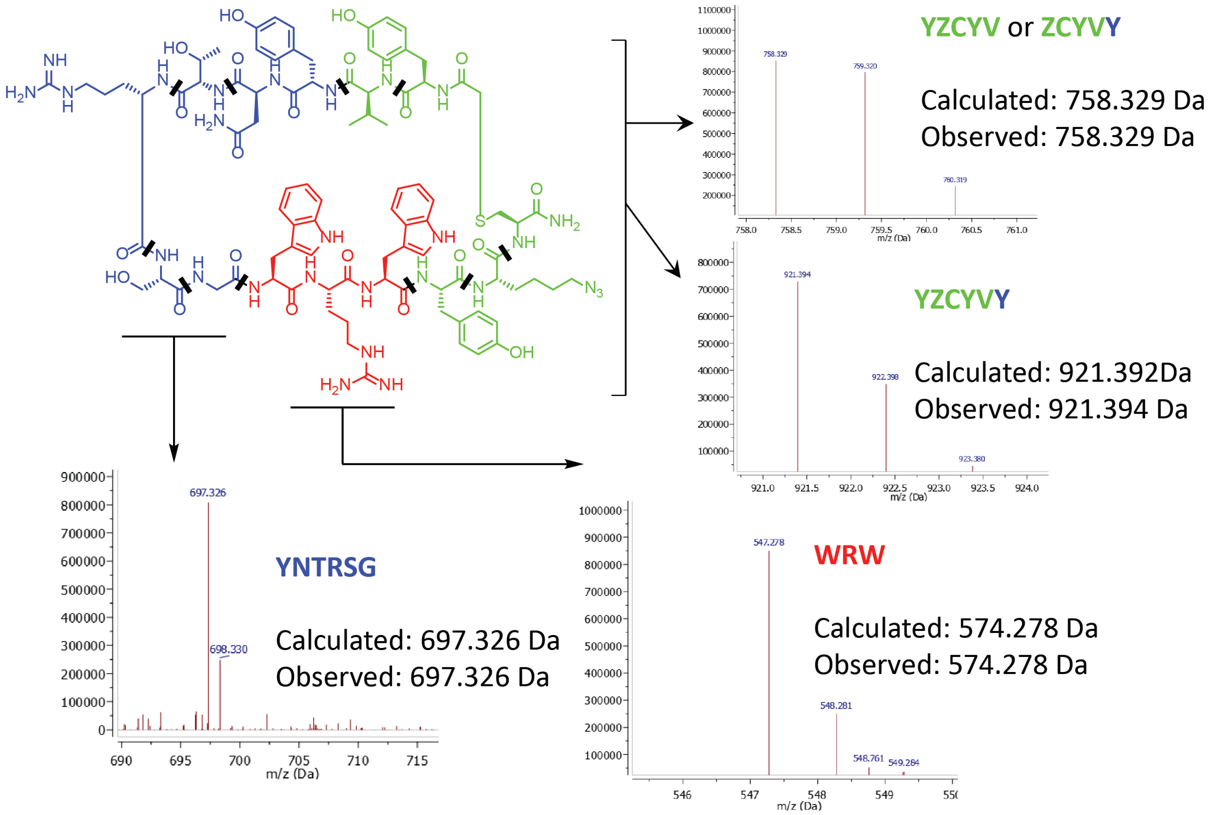


**Supplementary Figure 9.** Examples of MS spectra of the degradation products of CP2(T13Z) (entry 12) observed upon incubation with cell lysates.

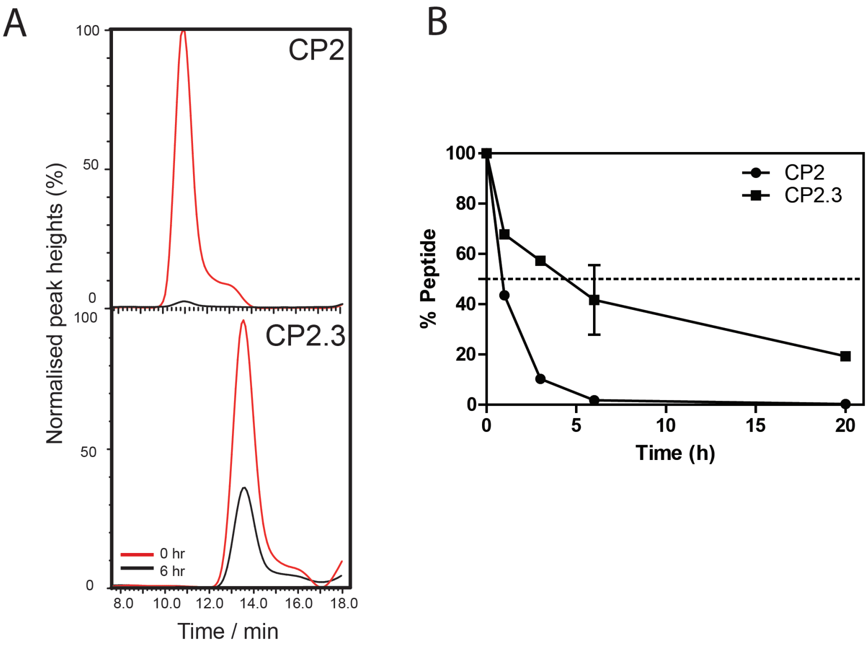


**Supplementary Figure 10. Proteolytic stability of CP2 and CP2.3 in HeLa cell lysate.** Peptides (10 µM) were incubated at 37°C in concentrated HeLa cell lysates, and relative stabilities of the two peptides over time were determined by quantitating the fraction of intact peptide by LCMS at each time interval. **(a)** LC trace at selected ion recording (SIR) channels corresponding to mass ions 948.11 *m/z* (CP2) and 967.4 *m/z* (CP2.3). **(b)** Half life ($t_{1/2}$) for CP2 was approximately 1hr, whereas CP2.3 was approximately 5 hrs (N=3, Avg +/- Std Dev).

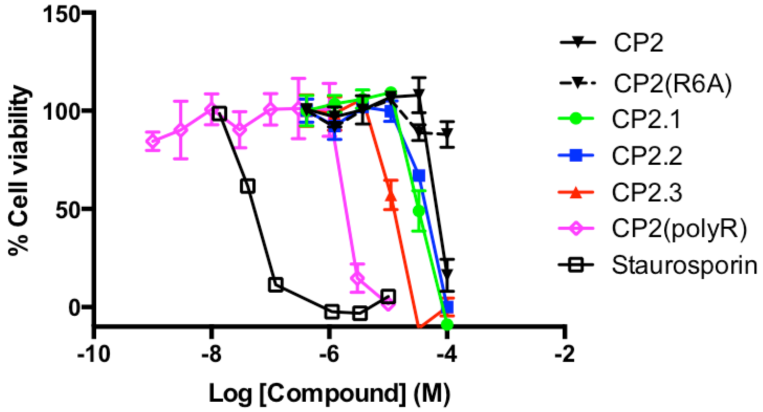


**Supplementary Figure 11. Cell proliferation assay of HeLa cells dosed with cyclic peptides.** Average +/- Stdev (N=3) are plotted.

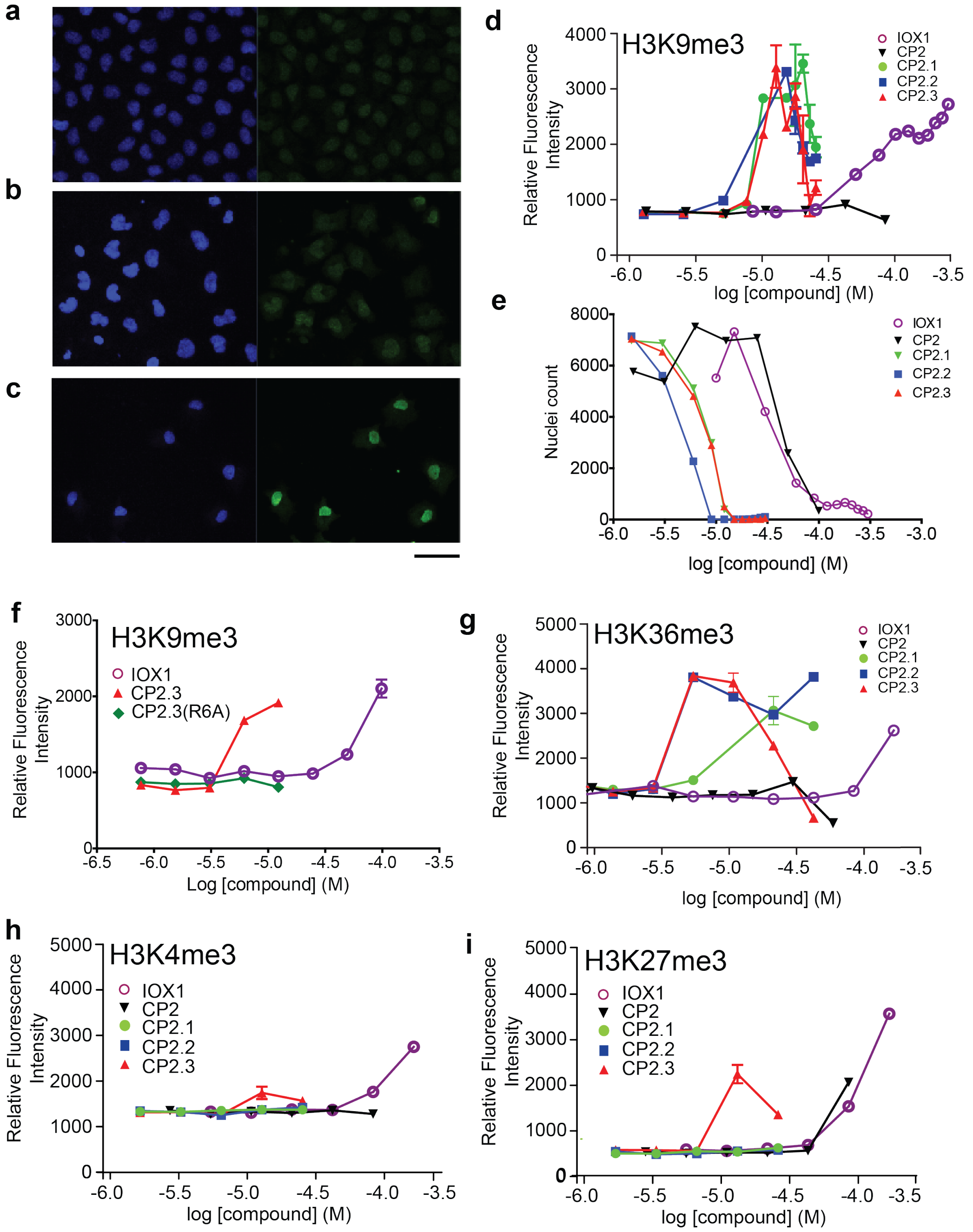


**Supplementary Figure 12. Immunofluorescence analysis of global histone methylation levels after 72hr dosing of cyclic peptides in HeLa cells. (a)-(c)** Immunofluorescence analysis of HeLa cells dosed with cyclic peptides for 72 hrs. Maximum intensity projected images of 3D confocal z-stacks showing nuclei stained with DAPI (blue) and anti-H3K9me3 antibody (green) for cells treated with (a) 1% DMSO, (b) CP2 (50 µM), (c) CP2.3 (12 µM) are shown. Note increased green fluorescence intensity with CP2.3 treated cells compared to DMSO and CP2 treated cells. Bar represents 50µm. Global H3K9me3 (d) and associated nuclei count for the experiment (e), (f) global H3K9me3 levels of CP2.3 relative to CP2.3(R6A) dosing (g) global H3K36me3 staining, (h) global H3K4me3 and (i) global H3K27me3 staining. An increase in fluorescence intensity, corresponding to increased H3K9me3/K36me3 levels was observed with increasing inhibitor (except for CP2) over certain concentration ranges. Average +/- s.e.m (n > 100 cells) are shown except at concentrations of CP2.1, CP2.2 and CP2.3 where n < 100 cells imaged (see (e)). A generic 2OG oxygenase inhibitor 5-carboxy-8-hydroxyquinoline (IOX1) which inhibits KDM4A at $EC_{50}$ ~ 100 µM in cells, was used as a control.[4]

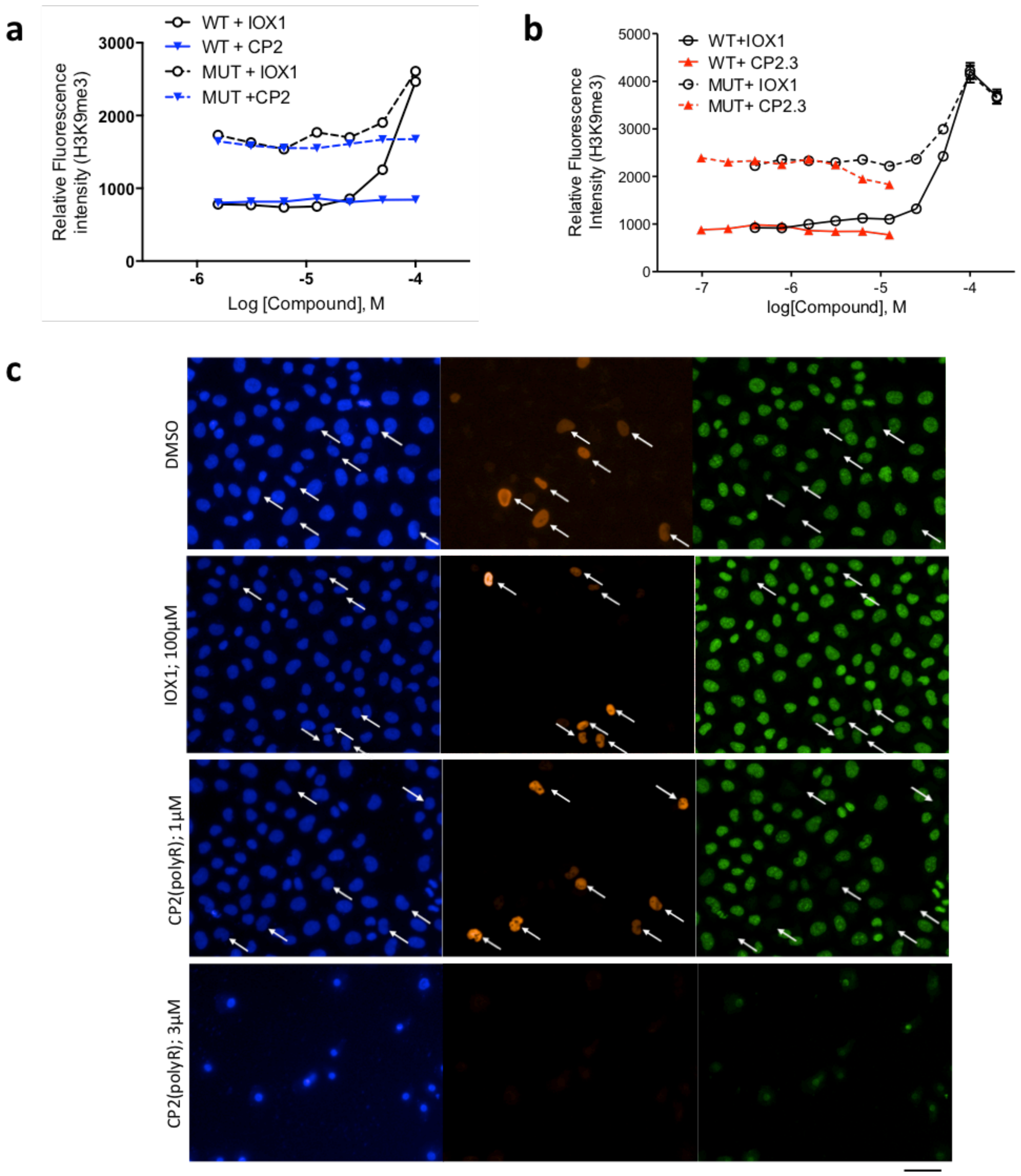


**Supplementary Figure 13**. Immunofluorescence analysis of ectopically expressed FLAG-KDM4A in HeLa cells dosed with cyclic peptides (24hrs). Dose-response analysis of nuclear staining of H3K9me3 of cells overexpressing FLAG-KDM4A wild type (WT) or catalytically inactive mutant H188A (MUT) with (a) CP2 and (b) CP2.3. Data shown, average +/- s.e.m (n > 100 cells). While an increase in H3K9me3 levels were observed for CP2.3 at 25 µM and 50 µM (data not shown), these data points had n < 10 transfected cells and were excluded. (C) Immunofluorescence images of HeLa cells dosed with CP2(polyR) and IOX1 (control). Representative images of nuclear staining (DAPI, blue), Flag-tagged staining (Flag-KDM4A, orange) and H3K9me3 (H3K9me3, green) are shown. Loss of H3K9me3 staining is observed for DMSO control and CP2(polyR)(1 µM) treated cells overexpressing KDM4A, while IOX1 treated cells overexpressing KDM4A show high levels of H3K9me3. No viable transfected cells are observed when dosed with CP2(polyR) at 3 µM. Bar represents 50 µm.

| | $IC_{50}$ (nM) | | | |
|---|---|---|---|---|
| | **CP2** | **CP2.1** | **CP2.2** | **CP2.3** |
| **KDM4A** | 42 | 29 | 100 | 110 |
| **KDM4B** | 33 | 151 | 2,520 | 1,020 |
| **KDM4C** | 29 | 15 | 274 | 69 |
| **KDM4D** | 6,270 | 6,440 | 5,250 | 6,240 |
| **KDM4E** | 9,200 | 19,100 | 18,400 | 11,000 |
| **KDM6B** | 6,800 | $>10^4$ | $>10^4$ | $>10^4$ |
| **KDM3A** | $>10^4$ | $>10^4$ | 7000 | $>10^4$ |
| **KDM5C** | $>10^4$ | 16,520 | $>10^4$ | $>10^4$ |

**Supplementary Table 1. Profiling of CP2 derived *N*-methylated cyclic peptides across human JmjC histone demethylases.**

| | KDM4A.Ni(II).CP2 | KDM4A.Ni(II).CP2 (R6Kme3) |
|---|---|---|
| **PDB acquisition codes** | 5LY1 | 5LY2 |
| **Data collection** | | |
| Beamline (Wavelength, Å) | DLS I04-1 (0.9173) | DLS I04-1 (0.9200) |
| Detector | Pilatus 2M | Pilatus 2M |
| Data processing | XDS[1], SCALA[2] | HKL2000[3] |
| Space group | $P2_1$ | $P2_1$ |
| Cell dimensions | | |
| $a$, $b$, $c$ (Å) | 57.27, 101.48, 140.33 | 58.31, 103.75, 142.37 |
| $\alpha$, $\beta$, $\gamma$ (°) | 90, 99.56, 90 | 90, 99.14, 90 |
| No. of molecules/ a.s.u. | 4 | 4 |
| No. reflections | 53856 | 62112 |
| Resolution (Å) | 49.34 – 2.50 (2.64 – 2.50)* | 48.67 - 2.43 (2.47 – 2.43)* |
| $R_{sym}$ or $R_{merge}$** | 0.071 (0.577)* | 0.138 (0.646)* |
| $I/\sigma I$ | 11.8 (2.1)* | 9.4 (2.0)* |
| Completeness (%) | 98.3 (98.3)* | 98.3 (95.0)* |
| Redundancy | 3.2 (3.3)* | 4.4 (3.9)* |
| CC (1/2) | 0.997 (0.704)* | 0.983 (0.671)* |
| Wilson $B$ value (Å$^2$) | 46.4 | 40.2 |
| **Refinement** | PHENIX[4], CNS[5] | PHENIX[4], CNS[5] |
| Resolution (Å) | 49.34 – 2.50 | 48.67 - 2.43 |
| $R_{factor}$/ $R_{free}$‡ | 0.178/ 0.205 | 0.219/ 0.245 |
| No. atoms▿ | | |
| Protein | A (2803), B (2765), C (2768), D (2779) | A (2810), B (2793), C (2778), D (2787) |
| Peptide | G (135) | E (136), F (74), G (134), H (74) |
| Metal/ion | Ni (4), Zn (4) ; Cl (3) | Ni (4), Zn (4), Cl (5) |
| Ligand (NOG) | - | A (10), B (10), C (10), D (10) |
| Water | A (97), B (74), C (124), D (103), E (7) | A (126), B (122), C (130), D (110), E (5), F (2), G (4), H (4) |
| B-factors▿ | | |
| Protein | A (53.8), B (57.5), C (45.8), D (51.5) | A (44.4), B (44.3), C (38.9), D (46.6) |
| Peptide | E (65.3) | E (53.7), F (59.0), G (53.8), H (68.3) |
| Metal/ion | Ni (A, 62.1; B, 63.9; C, 62.6; D, 68.1) Zn (A, 50.5; B, 50.4; C, 38.6; D, 46.0) Cl (A, 74.3 ; C, 66.2; D, 80.2) | Ni (A, 38.2; B, 40.4; C, 38.0; D, 43.7) Zn (A, 38.0; B, 40.2; C, 36.3; D, 46.4) Cl (A, 48.0 ; B, 55.7; C, 43.7; D, 55.0) |
| Ligand (NOG) | - | A (39.0), B (45.2), C (43.8), D (49.3) |
| Water | A (46.3), B (45.6), C (42.4), D (45.4), E (44.7) | A (41.2), B (42.6), C (38.4), D (41.3), E (44.3), F (43.2), G (44.0), H (43.5) |
| R.m.s deviations | | |
| Bond lengths (Å) | 0.004 | 0.005 |
| Bond angles (º) | 0.716 | 1.261 |

* Highest resolution shell is shown in parenthesis.

** $R_{sym} = \sum|I-\langle I\rangle|/\sum I$, where $I$ is the intensity of an individual measurement and $\langle I\rangle$ is the average intensity from multiple observations.

‡ $R_{factor} = \sum_{hkl}||F_{obs}(hkl)| - k\,|F_{calc}(hkl)||/ \sum_{hkl}|F_{obs}(hkl)|$ for the working set of reflections; $R_{free}$ is the $R_{factor}$ for ~5% of the reflections excluded from refinement.

▿ Indicates polypeptide chain

**Supplementary Table 2. Data collection and refinement statistics.**

| | Construct | Expression | [E] / nM | [2OG] / μM | [Fe(II)] / μM | Peptide / nM | Peptide sequence | Antibody | Source |
|---|---|---|---|---|---|---|---|---|---|
| KDM2A | 1-517 | *E.coli* | 25 | 10 | 10 | 20/80 | Biotin-H3K36(28-48)Me2 | Ab9048 | Abcam |
| KDM3A | 515-1317 | *Baculovirus* | 0.2 | 5 | 10 | 60 | H3(1 -21)K9Me2 -Biotin | Ab8896 | Abcam |
| KDM4A | 1-359 | *E.coli* | 5 | 10 | 1 | 30 | Biotin-H3(1-15)K9Me3 | Ab1220 | Abcam |
| KDM4B | 1-365 | *E.coli* | 5 | 10 | 1 | 30 | Biotin-H3(1-15)K9Me3 | Ab1220 | Abcam |
| KDM4C | 1-366 | *E.coli* | 5 | 10 | 1 | 30 | Biotin-H3(1-15)K9Me3 | Ab1220 | Abcam |
| KDM4D | 1-358 | *E.coli* | 5 | 10 | 1 | 30 | Biotin-H3(1-15)K9Me3 | Ab1220 | Abcam |
| KDM4E | 1-358 | *E.coli* | 2 | 10 | 1 | 30 | Biotin-H3(1-15)K9Me3 | Ab1220 | Abcam |
| KDM5C | 1-765 | *Baculovirus* | 0.5 | 40 | 10 | 30 | H3(1-21)K4Me3-Biotin | NEB9726 | Cell Signaling Technology |
| KDM6B | 1141-1641 | *E.coli* | 1 | 10 | 10 | 30 | Biotin -H3(14 -34)K27Me3 | 07-452 | Millipore |

**Supplementary Table 3. AlphaScreen assay parameters for 2OG oxygenases used for this study.** The table was dapted from [10]. Assays were performed at 2OG concentration near the 2OG Km values determined experimentally.[11] All enzymes were expressed and purified as described.[10-12]

**Supplementary methods:**

**Strain promoted Azide-Alkyne cycloaddition**

One equivalent of the azide (CP2(T13Z), peptide 12) was mixed with 1.5 – 2 eq of the bicyclo[6.1.0]non-4yne (BCN) conjugate (SynAffix B.V.) in $H_2O$/MeCN and shaken at 37 °C. Reaction times depend on the steric demand of the azide. Azidoalanine reacts in 24 h whereas the reaction with azidolysine was completed in about 5 h as determined by LC-MS analysis. The desired product was typically obtained in high yield (>90%) and the remaining BCN starting material was separated by HPLC purification (Agilent 1200 Series, Waters Sunfire column).

**Relative peptide stability assays in cell lysates**

HeLa cells (approximately 1 x $10^7$ cells) were washed twice with PBS, scraped and collected in 1.5ml PBS. Cells were centrifuged at 200g, 3 min at 4°C. The pelleted cells were lysed in buffer containing 20mM Tris-HCl (pH7.6), 137mM NaCl, 1% NP40, 10% glycerol and incubated for 15min at 4°C and centrifuged (14,000 g, 10 min, 4°C). The supernatant (cell lysate) was stored at -80°C until needed. Peptide samples were diluted in lysate (600 μL, equivalent to approximately 6 x $10^6$ cells) to 10 μM (1% DMSO final) and kept on ice. An initial 100 μL sample was taken as a T = 0 hr control and left on ice. The remainder of the sample (500 μL) was incubated at 37 °C, with 100 μL samples taken at 1hr, 3hr, 6hrs and 20hrs and kept on ice. Disposable SPE HyperSep C18 cartridges (ThermoScientific) were washed in 300 μL of elution buffer (60% MeCN, 0.1% formic acid in water), then twice with 200 μL wash buffer (0.5% MeCN, 0.1% formic acid in water) using a vacuum manifold. Samples were each loaded onto disposable SPE columns and washed twice in 200 μL wash buffer. The peptides were eluted in 700 μL elution buffer and concentrated down by vacuum centrifuge to 100 μL. The peptides (10 μL injection / sample) were analysed using reverse phase HPLC using a Grace Vydac analytical C18 column on a Waters Quattro CTC

instrument with a Waters autosampler. $H_2O$/acetonitrile with 0.1% formic acid was used as a mobile phase, an acetonitrile gradient (4% - 96%) was run over 23 min at 1ml/min, followed by 4% acetonitrile (30min total run). Spectra recorded were for the total ion count and Selected Ion recording (SIR) channels corresponding to the mass ions for CP2 (948.11 m/z) and CP2.3 (967.4 m/z) peptides. CP2 and CP2.3 standards were used for calibrating each LCMS runs, and concentrations of intact proteins were determined at each time-points against known peptide standards prepared in lysates in the same manner as the samples.

**Confocal and timelapse imaging**

For 3D timelapse confocal imaging, HeLa cells were seeded in 35mm glass-bottom dishes (World Precision Instruments) and cultured in regular culture medium supplemented with fluorescein-conjugated CP2 at a concentration of 10μM. 3D timelapse data were acquired using a LSM710 inverted confocal microscope (Carl Zeiss) with a 40x 1.3 N.A. phase contrast oil immersion lens. Fluorescein was excited with a 488nm argon-ion laser. A 3D dataset, with slices spaced 0.91μm apart, was acquired every 1.5 minutes for 6.5 h. During the entire timelapse experiment, the cells were kept at 37°C and 5% $CO_2$.

**Cell proliferation assay**

Cells were seeded (2,000 cells/well) in OptiMEM reduced serum supplemented with 0.5% FBS in 96well plates and dosed with compounds (1% DMSO final) the following day. After 18hrs, tetrazolium (MTT, CellTiter96Aqueous, (PerkinElmer)) was added and incubated for further 2 hrs. Absorbance at 490nm was measured and cell proliferation was normalised against cells treated with 1% DMSO and no cell controls.

**Immunofluorescence (IF) assay**

HeLa cells were maintained in OptiMEM reduced serum medium (Invitrogen) supplemented with 0.5% FBS and 1% Pen-Strep. Cells were seeded (1,500 per well) into a 96-well optical grade plate (Becton Dickinson) and left overnight to adhere. Cells were dosed with cyclic peptides and IOX1 (1% DMSO final) for 72h, with inhibitor supplemented media change every 24h. Cells were rinsed with PBS, fixed in 4% paraformaldehyde (20 minutes), and permeabilised with 0.5% TritonX-100 (10 min) in PBS. After 30min blocking (3% FBS in PBS), cells were incubated overnight with primary antibody (1:500) anti-histone H3 antibodies (K9me3 (Abcam, ab8988), K4me3 (Diagenode, pAB-003-050), K27me3 (Millipore, 07-449), K36me3 (Abcam, ab9050) and further incubated with the secondary antibody (1:500) goat anti-rabbit Alexafluor 488 (Invitrogen) (1h). Cell nuclei were stained with 4',6-diamidino-2-phenylindole (DAPI) (1:1000), (Invitrogen). Cells were washed in PBS three times after each incubation. For IF assays ectopically overexpressing FLAG-KDM4A (WT and catalytically inactive mutant (H188A), Mut), cells were transfected for 4hrs prior to dosing with compounds for 24hrs as previously described.[4] Cells were washed, fixed, permeablised and blocked as above. Monoclonal mouse anti-FLAG primary antibody (F1804, Sigma at 1:500) was used for FLAG-staining with the secondary antibody (1:500) goat anti-mouse Alexafluor 594 (Invitrogen).

**Image acquisition and analysis**

The Pathway (Beckton Dickinson), an automated high-content imaging machine, was used to image immunostained cells in a 96-well plate configuration. For each well, the system acquired a 3-by-2 tile-scanned images for Alexafluor 488 and DAPI. During analysis, the Pathway software used the DAPI staining to identify nuclei as regions-of-interests (ROI). For each nucleus, the software extracted the average intensity of the histone staining, followed by the average intensity of all the nuclei in that particular well. The average intensity for each

well was plotted against the corresponding concentration to obtain a dose response curve, and analysed using GraphPad Prism 5. For IF assays using KDM4A overexpressed cells, the average intensity of H3K9me3 was determined for the population of cells expressing FLAG-KDM4A.

**Supplementary References**


1 Hipolito, C. J. & Suga, H. Ribosomal production and in vitro selection of natural product-like peptidomimetics: the FIT and RaPID systems. *Current opinion in chemical biology* **16**, 196-203, doi:10.1016/j.cbpa.2012.02.014 (2012).

2 Chowdhury, R. *et al.* The oncometabolite 2-hydroxyglutarate inhibits histone lysine demethylases. *EMBO reports* **12**, 463-469, doi:10.1038/embor.2011.43 (2011).

3 Williams, S. T. *et al.* Studies on the catalytic domains of multiple JmjC oxygenases using peptide substrates. *Epigenetics* **9**, 1596-1603, doi:10.4161/15592294.2014.983381 (2014).

4 King, O. N. *et al.* Quantitative high-throughput screening identifies 8-hydroxyquinolines as cell-active histone demethylase inhibitors. *PloS one* **5**, e15535, doi:10.1371/journal.pone.0015535 (2010).

5 Kabsch, W. Integration, scaling, space-group assignment and post-refinement. *Acta crystallographica. Section D, Biological crystallography* **66**, 133-144, doi:10.1107/S0907444909047374 (2010).

6 Winn, M. D. *et al.* Overview of the CCP4 suite and current developments. *Acta crystallographica. Section D, Biological crystallography* **67**, 235-242, doi:10.1107/S0907444910045749 (2011).

7 Otwinowski, Z. & Minor, W. Processing of X-ray diffraction data collected in oscillation mode. *Method Enzymol.* **276**, 307-326, doi:Doi 10.1016/S0076-6879(97)76066-X (1997).

8 Adams, P. D. *et al.* PHENIX: a comprehensive Python-based system for macromolecular structure solution. *Acta crystallographica. Section D, Biological crystallography* **66**, 213-221, doi:10.1107/S0907444909052925 (2010).

9 Brunger, A. T. *et al.* Crystallography & NMR system: A new software suite for macromolecular structure determination. *Acta crystallographica. Section D, Biological crystallography* **54**, 905-921 (1998).

10 Rose, N. R. *et al.* Plant Growth Regulator Daminozide Is a Selective Inhibitor of Human KDM2/7 Histone Demethylases. *Journal of medicinal chemistry* **55**, 6639-6643 (2012).

11 Hopkinson, R. J. *et al.* 5-Carboxy-8-hydroxyquinoline 1 is a broad spectrum 2-oxoglutarate oxygenase inhibitor which causes iron translocation. *Chemical Science*, in press, doi:10.1039/c3sc51122g (2013).

12 Hillringhaus, L. *et al.* Structural and evolutionary basis for the dual substrate selectivity of human KDM4 histone demethylase family. *The Journal of biological chemistry* **286**, 41616-41625, doi:10.1074/jbc.M111.283689 (2011).
